# Smartphone Usage Patterns and Sleep Behavior in Demographic Groups: Retrospective Observational Study

**DOI:** 10.2196/60423

**Published:** 2025-07-03

**Authors:** Ting Wang, Anja Seiger, Alexander Markowetz, Ionut Andone, Konrad Błaszkiewicz, Thomas Penzel

**Affiliations:** 1 Interdisciplinary Center of Sleep Medicine Charité – Universitätsmedizin Berlin Berlin Germany; 2 Department of Computer Science University of Bonn Bonn Germany

**Keywords:** smartphone usage patterns, nocturnal smartphone inactivity, demographics, sleep health, smartwatch

## Abstract

**Background:**

Although previous studies have examined the relationship between smartphone usage and sleep disorders, research on demographic differences in smartphone usage and nocturnal smartphone inactivity patterns remains limited. This study introduces “nocturnal smartphone inactivity duration” as a proxy indicator to address the limitation of lacking direct sleep data and to further investigate the association between smartphone usage patterns and sleep characteristics.

**Objective:**

This study aimed to investigate demographic differences and relationships between daily smartphone usage and nocturnal smartphone inactivity patterns.

**Methods:**

We conducted a retrospective analysis of data collected from the Murmuras app from January 1, 2022, to December 31, 2022. A total of 1074 participants were included, categorized by gender, age, highest degree, employment status, and smartphone usage purpose. All participants consented to participate in the study through the app. To explore the relationship between smartphone usage and nocturnal smartphone inactivity, we first calculated each participant’s daily smartphone usage duration (including app usage) and duration of nocturnal smartphone inactivity; then, we assessed the normality and homogeneity of variance tests within each demographic category. Based on the results, the Kruskal-Wallis tests were applied to potentially identify differences between groups. Finally, correlation and regression analyses were conducted to explore associations between smartphone usage and nocturnal smartphone inactivity.

**Results:**

The findings revealed distinct patterns of smartphone use across demographics. Participants predominantly used smartphones for social contact (average daily usage duration=1.52 h) and recreational activities (average daily usage duration=1.08 h) through apps like Facebook and YouTube. Frequent users, especially of social media and entertainment, often increased their phone usage at night. Female participants used their phones more frequently, mainly for digital shopping and social interactions, whereas male participants used phones more at nighttime (*P*<.001). Both younger users and non–full-time employees engaged more in activities such as gaming and chatting (*P*<.01 for those comparisons). Higher education was correlated with lower use (*P*<.001). Those using smartphones for work-related purposes generally decreased their phone usage after work (*P*<.05 for those comparisons). Correlation and regression analyses of smartphone usage duration and nighttime inactivity across groups indicated that only a small subset of groups exhibited significant positive correlations, a moderate number displayed significant negative correlations, and the majority showed no significant correlation.

**Conclusions:**

This study underscores the significant association between demographic factors and smartphone usage patterns, including nocturnal inactivity patterns. Female individuals, young people, individuals with lower educational qualifications, and those who were unemployed demonstrated higher smartphone usage. Frequent engagement with social media and leisure apps was particularly pronounced during nighttime hours, a behavior that may contribute to disruptions in sleep patterns. These findings underscore the need for targeted interventions addressing excessive smartphone use, particularly at night, to mitigate its potential adverse effects on sleep.

## Introduction

According to Statista, by 2024, the global number of smartphone users is set to reach an astounding 6.93 billion, equating to 85.74% of the world’s population. This figure is expected to soar beyond 7.7 billion by 2028 [1]. Smartphones provide seamless internet access and the ability to run a diverse range of apps, thereby catering to a variety of needs such as communication, internet browsing, gaming, organizing schedules, video streaming, and social networking [2,3]. Moreover, smartphones have emerged as an efficient platform for delivering cost-effective, scalable, and high-quality health interventions to diverse populations [4]. Examples include SMS reminders for childhood vaccinations in Ethiopia [5] and tools for screening and managing sleep disorders [6].

However, smartphone technology also brings with it a plethora of physical and psychological challenges. Physical ailments include issues such as head and neck discomfort [7]; tendonitis [8,9]; and eye-related issues like eye strain, blurred vision, and acute acquired esotropia [10]. Psychologically, excessive smartphone use has been linked to concerns such as anxiety, depression [11], “smartphone addiction” [12], “smartphone phobia” (fear of being unable to use a smartphone) [13], and various sleep disturbances [14]. Prolonged smartphone use, particularly at night, has been shown to disrupt sleep patterns, delay sleep onset, shorten total sleep duration, and impair sleep quality [15-17]. Multiple studies have identified a significant relationship between nighttime smartphone use and sleep disruption [18,19]. Notably, research by Wood et al [19] highlighted that smartphone use before bedtime can interfere with the rhythm of melatonin secretion and alter sleep electroencephalogram patterns. Additionally, presleep smartphone use may provoke cognitive arousal or emotional instability, further exacerbating insomnia [20,21].

Adequate sleep is fundamental to maintaining overall health and well-being. Adolescents require 8-10 hours and adults need 7-8 hours nightly [22,23]. Insufficient sleep has been linked to adverse outcomes such as daytime sleepiness, cognitive impairment, reduced work efficiency, and an elevated risk of cardiovascular diseases [24,25]. Given the ubiquity of smartphones and their prevalent nighttime use, understanding the intricate relationship between smartphone use and sleep is critical.

Various advanced technologies have been developed to monitor and analyze sleep behavior, such as Fitbit, Withings, and Apple Watch. These devices use embedded accelerometers, temperature sensors, and heart rate monitors to provide accurate sleep tracking data [26]. Although such tools enable health care professionals to gain insights into sleep behaviors, their high cost and dependence on additional hardware limit their accessibility for broader populations. In contrast, smartphones offer a more practical and cost-effective alternative for investigating sleep patterns due to their widespread availability and daily integration into users’ lives. A Pew Research Center survey found that 65% of smartphone users (and an impressive 90% of teenagers) keep their phones on or near their beds while sleeping, with many using smartphones as alarm clocks [27]. This deep integration into daily routines underscores the potential of smartphones as a powerful tool for understanding sleep behavior.

Behavioral research supports the concept of “nocturnal smartphone inactivity” as a reliable proxy for assessing sleep behavior. Most individuals cease using their smartphones once they fall asleep, making the duration of nocturnal smartphone inactivity an effective indirect measure of sleep patterns. Several studies validate this approach. Ciman and Wac [26] found that smartphone screen on and off patterns could estimate sleep duration, sleep onset, wake times, and even sleep deprivation patterns, with only a 7% margin of error compared to smartwatch data. In 2019, a study conducted in the Netherlands reported a strong correlation (*R*^2^=0.9) between smartphone touch data and traditional sleep measurement methods, enabling accurate predictions of sleep onset and wake times [28]. Cuttone et al [29] showed that changes in smartphone interaction frequency could infer individual sleep patterns, highlighting the close association between smartphone activity and sleep behaviors.

These findings collectively underscore the validity of nighttime smartphone usage patterns as a reliable indicator of sleep duration and behavior. Although existing research has primarily focused on the relationship between post–lights-out smartphone usage and sleep disorders, the underlying factors associated with nighttime smartphone usage remain insufficiently explored. To address this research gap, this study retrospectively analyzed smartphone usage data from 1074 individuals across diverse demographic groups, collected through the Murmuras app. This study was conducted in three phases: (1) collecting demographic characteristics and smartphone usage data, (2) comparing smartphone usage patterns among different demographic groups, and (3) exploring correlation between daily smartphone use and nocturnal inactivity.

To operationalize sleep behavior, this study referenced international sleep health standards. According to them, adults who sleep less than 6 hours per night are generally classified as experiencing “short sleep,” a condition strongly associated with various health risks, including cardiovascular diseases and cognitive impairments [30]. In this study, the duration of nocturnal smartphone inactivity was considered an indirect proxy variable for reflecting participants’ sleep behaviors. Specifically, the presence of continuous nocturnal smartphone inactivity of 6 hours or more was adopted as a criterion for adequate sleep duration, while nocturnal smartphone inactivity of less than 6 hours was classified as potential sleep deprivation.

This study aims to (1) examine the differences in smartphone usage patterns across demographic groups and (2) evaluate the relationship between daily smartphone use and nocturnal smartphone inactivity, providing indirect evidence for understanding the broader connection between smartphone behaviors and sleep patterns. The study introduces “nocturnal smartphone inactivity duration” as an innovative proxy for assessing sleep behavior. This approach aligns with established theoretical and empirical frameworks, offering both scientific rigor and practical applicability. Even in the absence of direct sleep data, this proxy effectively captures the relationship between smartphone usage patterns and sleep, laying a robust foundation for interdisciplinary research at the intersection of behavior and health.

## Methods

### Study Design

This study follows the STROBE (Strengthening the Reporting of Observational Studies in Epidemiology) guidelines for reporting observational research. It aims to investigate user behavioral patterns and their potential associations with sleep behaviors by analyzing the relationship between smartphone usage patterns and nocturnal smartphone inactivity.

The analysis was conducted in 3 phases. First, the demographic characteristics and smartphone usage of all 1074 participants were analyzed. Second, participants were stratified into groups based on gender, age, highest education level, employment status, and primary type of smartphone usage. Statistical tests were used to identify differences in smartphone usage patterns across these demographic groups. Finally, correlation and regression analyses were conducted to explore the association between smartphone usage and nocturnal smartphone inactivity.

### Study Equipment

We carried out a retrospective, siteless study using data provided by the Murmuras app, in continuation of the Menthal project [31]. All data were securely stored on the Murmuras platform, with publishing rights retained by all authors, irrespective of the study outcomes.

The Murmuras app is specifically designed to capture detailed smartphone usage data, supported by a robust back-end web platform, enabling extensive and in-depth analysis. Beyond monitoring smartphone app usage, the Murmuras app tracks a wide range of device-level interactions, including screen on and off events, device unlocking, frequency of startup and shutdown, SMS activity (sent and received), and incoming and outgoing calls. In addition to behavioral data, the Murmuras app gathers user feedback on personality traits, demographic characteristics, and emotional states via structured questionnaires.

### Study Population

Participants were recruited through the Murmuras app between January 1, 2022, and December 31, 2022. Users who installed the app from the Google Play Store and consented to data sharing were included. The study used passively collected smartphone usage data, which were analyzed retrospectively.

### Ethical Considerations

This study was conducted in accordance with ethical guidelines, ensuring the protection of participant privacy and data confidentiality. The original data collection was approved by the Ethics Committee of the University of Bonn (approval #13-05-31). All participants provided informed digital consent via the mobile app prior to the initial data acquisition. The dataset includes smartphone usage behavior data only and does not contain any personally identifiable information.

As this study involved a secondary analysis of fully anonymized data, no additional institutional review board approval was required. This complies with the General Data Protection Regulation and the ethical standards outlined by the *Arbeitskreis Medizinischer Ethik-Kommissionen* [32]. Permission to access and use the dataset for secondary research purposes was obtained from the University of Bonn and the original data custodians. Participants did not receive any form of compensation for their involvement in the original study.

### Data Collection

Data collection was facilitated through the Murmuras app, which confirmed eligibility, secured participant consent, and gathered phone usage data. Upon consent, participants downloaded the app and used a unique, anonymous ID code provided via the link for initial access.

The study encompassed a standard demographic survey (age, gender, education, occupation, etc) and recorded data on app usage (such as app name, usage duration, and the sequence of usage) and device interaction data (such as screen on and off events and device power cycles). All collected data were systematically formatted and organized into CSV files.

Data for this study were collected retrospectively using the Murmuras app, which is primarily marketed and widely used in Germany. As a result, the majority of participants were from Germany.

### Key Indicators

The key indicators were as follows:

- Average daily smartphone usage duration and frequency: This metric captures the daily duration and frequency of smartphone usage for each participant, including the use of various apps.
- App usage patterns by category: Using Murmuras app data, we have categorized all recorded apps into 6 functional groups, based on prior research and app store classifications [33]:
  Type A—network app: these apps serve the purpose of acquiring information through network searches and related functions, such as Google, Firefox, and Duolingo.
  Type B—leisure and entertainment: this category encompasses apps that revolve around entertainment activities, such as gaming, video watching, and reading.
  Type C—functional: apps falling under this classification are utility-based and offer practical functions like clocks, calculators, and more.
  Type D—emotional: these apps are primarily used for social engagement, including activities like chatting and messaging.
  Type E—monetary: apps in this category are designed for financial transactions and various money-related activities.
  Type unknown: this category includes apps with unclear or unspecified primary functions.
- Nocturnal smartphone inactivity duration: The longest continuous period of nonuse of the smartphone at nighttime, defined as the time between 19:00 and 06:00 the following day.

### Statistical Analysis

The statistical analysis entailed calculating the daily duration of smartphone usage, daily duration and frequency of different smartphone app usage, and duration of nocturnal smartphone inactivity. Normality and homogeneity of variance tests were first conducted to evaluate the distribution of smartphone usage patterns within each demographic group. Based on the test results, Kruskal-Wallis tests were applied to identify differences between groups, followed by the Dunn post hoc test for pairwise comparisons. Statistical significance was determined using *P* values, and all indicators were reported as medians with IQRs (first and third quartiles). Correlation and regression analyses were conducted to explore relationships between smartphone usage and nocturnal smartphone inactivity. Results were presented through correlation coefficients, regression coefficients, intercepts, *P* values, and *R*^2^ values.

Data categorized as “Other,” “Prefer not to answer,” or “Unknown” were excluded from the analysis to ensure reliability and validity. The study included 1074 participants, classified based on gender, age, employment status, education level, and purpose of smartphone use—variables answered by the vast majority of respondents. Other demographic variables, such as relationship status, living area, and income level, were excluded due to high non-response rates, with over 45% (n=470) of participants opting not to answer. This focus on core demographic factors ensured robust and reliable statistical results. All statistical analyses were encoded and analyzed using RStudio (2023.06.2 Build 561; Posit).

## Results

### Participant Characteristics and Smartphone Usage Patterns

This research encompassed 1074 smartphone users, with most (n=724, 67.4%) of the sample being female. Participants were from 39 countries, with the majority residing in Germany (91.5%, n=983). The mean age of participants was 27.9 (SD 9.5) years.

In total, 59.3% (n=637) of participants held a high school degree or equivalent educational background, while 49.8% (n=535) were students. The data revealed that smartphones were not only primarily used for social media and leisure entertainment but also frequently for managing daily affairs. The average daily duration of smartphone usage was 3.54 hours. A total of 91.9% (n=987) of participants did not use their phones for more than 6 hours during nighttime (Tables 1 and 2).

**Table 1:** Participants demographics and smartphone usage characteristics.

Group category		Participants (N=1074), n (%)
**Gender**		
	Female	724 (67.4)
	Male	350 (32.6)
**Age range (years)**		
	<18	8 (1)
	≥18 to <35	895 (83.3)
	≥35 to <60	161 (14.9)
	≥60	10 (1)
**Highest degree**		
	Doctorate	11 (1)
	Master’s degree	116 (10.8)
	Bachelor’s degree	189 (17.6)
	Secondary education	96 (9)
	High school degree or equivalent	637 (59.3)
	No formal qualification	10 (1)
	Other	12 (1)
	Prefer not to answer	3 (0.3)
**Smartphone use type**		
	Both equally	139 (12.9)
	Mainly private	390 (36.3)
	Mainly work	14 (1)
	Private only	524 (48.8)
	Work only	7 (1)
**Has children**		
	No	471 (43.9)
	Yes	127 (11.8)
	Prefer not to answer	476 (44.3)
**Household net income (€)^a^**		
	10,000-20,000	25 (2)
	100,000-150,000	3 (0.3)
	20,000-30,000	12 (1)
	90,000-100,000	2 (0.2)
	Less than 10,000	34 (3)
	Prefer not to answer	998 (92.9)
**Nationality (country)**		
	Germany	983 (91.5)
	Other countries	91 (8)
**Employment status**		
	In education	535 (49.8)
	Unemployed or job seeking	20 (2)
	Part-time	149 (13.9)
	Full-time	267 (24.9)
	Self-employed	41 (4)
	Homemaker	14 (1)
	Retired	17 (2)
	Other	23 (2)
	Prefer not to answer	8 (1)
**Relationship status**		
	Divorced or separated	17 (2)
	In a relationship or married	243 (22.6)
	Registered partnerships	109 (10.2)
	Single	218 (20.3)
	Widowed	8 (1)
	Prefer not to answer	479 (44.6)
**Personal net income (€)** ^a^		
	10,000-20,000	141 (13.1)
	100,000-150,000	1 (0.1)
	20,000-30,000	128 (11.9)
	Less than 10,000	177 (16.5)
	Prefer not to answer	627 (58.4)
**Lived in self-owned property**		
	No	119 (11.1)
	Yes	16 (1)
	Prefer not to answer	939 (87.4)

^a^€ 1= US $1.12.

**Table 2:** Overview of smartphone usage and nocturnal inactivity patterns.

Category and subcategory				Value
**All participants (n=1074)**				
	Daily duration of smartphone usage (h)			3.54
	**Daily duration of smartphone app usage (h)**			
		Type A	0.67	
		Type B	1.08	
		Type C	0.29	
		Type D	1.52	
		Type E	0.13	
		Type unknown	0.07	
	**Proportion of daily** **smartphone app** **usage (%)**			
		Type A	14.8	
		Type B	8.3	
		Type C	38.9	
		Type D	32.1	
		Type E	1.2	
		Type unknown	4.6	
**Participants with** **nocturnal** **smartphone** **inactivity** **exceeding 6 hours (n=** **987)**				
	Daily nocturnal inactivity duration of days with ≥6 hours of nocturnal inactivity (h)			8.72
	Proportion of days with ≥6 hours of nocturnal inactivity (%)			65.5

### Correlations of Smartphone Usage Patterns With Demographic Characteristics

#### Gender Differences

Significant gender differences were observed in the usage durations of types C, D, and E. Female participants spent more time on chat apps (type D) and consumer-related apps (type E; *P*<.001 for those comparisons). Notably, female participants exhibited longer durations of smartphone usage, averaging 3.49 hours per day, compared to male participants (*P*<.001).

#### Age Differences

Age was closely associated with the usage durations of types B, C, and D. These differences were particularly pronounced in entertainment apps (type B) and social apps (type D; *P*<.001 for both comparisons). Younger participants (3.01-3.46 h) had higher smartphone usage duration compared to older participants (1.85 h; *P*<.001 for those comparisons).

#### Education Level Differences

Educational qualifications were strongly associated with smartphone usage patterns, particularly for types A, B, and D. Participants with a doctoral degree reported the lowest smartphone usage (2.26 h), followed by those with a master’s degree (2.60 h), a secondary education (2.91 h), a bachelor’s degree (3.37 h), and a high school degree or equivalent (3.58 h). Those without any formal qualification reported the highest usage at 4.03 hours (*P*<.001 for those comparisons).

#### Employment Status Differences

Employment status showed notable variations in the usage duration of types B, D, and E among employment categories, such as full-time employees, homemakers, and retirees (*P*<.001, *P*<.001, and *P*=.003, respectively). Participants in education reported the high smartphone usage (3.68 h), followed by individuals who were unemployed and job seeking (4.46 h), homemakers (4.28 h), full-time workers (3.20 h), part-time workers (2.85 h), and self-employed individuals (2.46 h). Retired participants had the lowest median usage (1.48 hours; *P*<.001 for those comparisons).

#### Smartphone Usage Type Differences

The primary purpose of smartphone usage was significantly linked to variations in the usage duration across all app categories (A, B, C, D, and E; *P*<.001, *P*<.001, *P*<.001, *P*<.001, and *P*=.007, respectively). Participants using their phones primarily for work activities demonstrated higher engagement with tools such as email, calendar, and document management apps. Participants who used smartphones mainly for work had the shortest usage (0.43 h), followed by those who used them exclusively for work (0.17 h). Participants who used smartphones mainly for private purposes (3.52 h), exclusively for private use (3.35 h), or equally for both work and private purposes (3.17 h) reported longer usage durations (detailed data are provided in Multimedia Appendices 1-6).

### Correlations of Nocturnal Smartphone Inactivity With Demographic Characteristics

Initially, we analyzed the periods of nocturnal smartphone inactivity among participants. Participants were categorized into 2 groups based on results: group 1 comprised participants with no nightly smartphone inactivity period of 6 hours or longer, and group 2 comprised participants who recorded at least one nocturnal smartphone inactivity period of 6 hours or longer.

#### Participants With Less Than 6 Hours of Nocturnal Smartphone Inactivity (Group 1)

Among the 1074 participants, 86 (8%) individuals had a nocturnal smartphone inactivity duration of less than 6 hours. The proportion varied across demographic groups.

By gender, 12% (41/350) of male participants had nocturnal inactivity below 6 hours, compared to 6% (45/724) of female participants, with a statistically significant difference (*P*=003).

Age group analysis showed that the proportion was highest among participants younger than 18 years (1/8, 13%), followed by those aged 18 to <35 years (75/895, 8%), 35 to <60 years (10/161, 6%), and 60 years and older (0/10, 0%), with no statistically significant difference observed (*P*=.54).

In terms of educational attainment, the proportion of participants with low nocturnal inactivity ranged from 0% (0/10) among those with no formal qualification to 9% (1/11) among those with doctorate or master’s degrees. Other groups included those with bachelor’s degrees (16/96, 8%), high school degree or equivalent (53/637, 8%), and secondary education (6/96, 6%). However, these differences were not statistically significant (*P*=.97).

Employment status was significantly associated with nocturnal inactivity duration (*P*=.03). The highest proportion was observed among self-employed participants (7/41, 17%), followed by those in education (50/535, 9%), part-time workers (12/149, 8%), homemakers (1/14, 7%), retired individuals (1/17, 6%), and full-time employees (11/267, 4%). None of the participants who were unemployed and job seeking (0/20, 0%) had nocturnal inactivity below 6 hours.

Regarding smartphone use type, 29% (2/7) of participants who used their phones exclusively for work had low nocturnal inactivity, compared to 11% (15/139) among those who used both equally, 8% (43/524) for private-only users, 7% (26/390) for mainly private users, and 7% (1/14) for mainly work users. These differences were not statistically significant (*P*=.15). (Multimedia Appendix 7).

#### Participants With More Than 6 Hours of Nocturnal Smartphone Inactivity (Group 2)

Among participants with at least one night of smartphone inactivity exceeding 6 hours, variations in both the median duration and the proportion of such nights were observed across demographic subgroups.

For gender, male participants exhibited a slightly longer median nocturnal inactivity duration (8.34 h) compared to female participants (8.28, IQR 7.77-8.85 h), though the difference was not statistically significant (*P*=.08). However, female participants had a significantly higher proportion of nights with over 6 hours of inactivity (73.68%) than male participants (64.81%; *P*<.001).

Significant differences were also observed by age group (*P*<.001). Older adults (≥60 years) had the longest median duration (10.81 h) and the highest proportion (94.58%). In contrast, participants younger than 18 years had a high duration (8.92 h) but the lowest proportion (46.20%). Middle-aged adults (35-59 years) showed both high duration (8.30 h) and proportion (80.93%), followed by young adults (18-34 years) with 8.28 hours and 69.82%.

For educational attainment, median durations ranged from 8.18 to 8.58 hours with no statistically significant difference (*P*=.24). However, the proportion of nights over 6 hours differed significantly (*P*=.001), with the highest values observed among those with a doctorate (81.27%) and master’s degree (79.13%), while individuals with no formal qualification had the lowest (50.83%).

Across employment status, both duration (*P*<.001) and proportion (*P*=.002) showed significant variation. Retired participants had the longest inactivity (9.91 h) and highest proportion (86.02%), while those who were unemployed and job seeking had shorter durations and the lowest proportion (33.33%). Full-time and part-time workers reported median durations of 8.15 and 8.22 hours, and proportions of 74.45% and 76.30%, respectively. Students and homemakers had similar values, while self-employed participants showed moderate durations (8.39 h) and high proportions (72.73%).

Regarding smartphone use type, the longest durations were found among participants who used their phones mainly for work (14.42 h) or only for work (13.94 h; *P*<.001). However, differences in the proportion of nights with >6 hours of inactivity were not statistically significant across use types (*P*=.17), ranging from 57.14% to 74.71% (Multimedia Appendices 7-9).

### Correlation and Regression Analyses of Smartphone Usage and Nocturnal Inactivity

#### Associations Between Smartphone App Usage and Nocturnal Inactivity

Analyses generally showed low and nonsignificant correlations between app usage and nocturnal smartphone inactivity. However, several exceptions were observed.

Among participants with doctoral degrees, a positive correlation was identified between the usage of type A (eg, professional or academic tools) and nocturnal smartphone inactivity (*r*=.89; *P*=.002). In contrast, the secondary education group exhibited a weak negative correlation across multiple app types (A, B, C, D, and E; *P*<.05 for those comparisons), while participants with no formal education showed a moderately negative correlation specifically for type D apps (*r*=–.70; *P*=.02). The unemployed or job-seeking group demonstrated a moderate positive correlation noted for type B apps (*r*=.55; *P*=.01). In the homemaker group, a strong negative correlation was observed for type D app (*r*=–.66; *P*=.01). Similarly, work-focused participants, who primarily or exclusively used smartphones for professional purposes, exhibited strong negative correlations across the usage of app types A, C, and D (*P*<.05 for those comparisons; refer to Multimedia Appendices 10-12 for additional details).

#### Associations Between Smartphone Usage and Nocturnal Inactivity

Among smartphone users aged 18-60 years, a weak negative correlation was observed (*P*=.009 and *P*=.04, respectively), indicating that longer usage duration was modestly associated with shorter nocturnal smartphone inactivity periods. In the secondary education group, the correlation was a stronger negative correlation coefficient (*r*=–.42; *P*<.001). Employment status further showed variability in this relationship, with the full-time employee, homemaker, and retired groups all demonstrating significant negative correlations. Notably, the homemaker group exhibited the strongest correlation (*r*=–.66; *P*=.02), closely followed by the retired group (*r*=–.64; *P*=.007). Additionally, participants who primarily used their smartphones for work-related purposes displayed exceptionally strong negative correlations (*r*=–.89 to –.96; *P*<.05 for those comparisons), emphasizing the substantial association of work-focused smartphone usage (see Multimedia Appendices 10 and 13 for detailed data).

## Discussion

### Principal Findings

This study demonstrated that smartphone usage patterns were significantly associated with factors such as gender, educational attainment, employment status, and usage purpose. Generally, female participants showed a higher frequency of phone usage compared to male participants, predominantly for online shopping and social interaction apps. Male participants were more likely to engage with their smartphones during nighttime hours. Younger participants exhibited more extensive use of smartphones, primarily for playing games, watching videos, and chatting with friends. Older individuals, meanwhile, showed lower levels of smartphone engagement. Participants with higher levels of education generally spent less time on smartphones and were more likely to use information acquisition apps, such as Google and news platforms. Individuals not employed full-time, including part-time employees or homemakers, often used their smartphones more intensively, especially at night, mainly for activities such as gaming, video streaming, and social interactions. Additionally, homemakers displayed a preference for financial apps, such as those used for stock trading, financial management, and online shopping. Conversely, individuals who primarily used smartphones for work-related purposes had lower usage compared to those who used them for personal purposes. Their smartphone activities were predominantly focused on work-related tasks, including communicating with colleagues, sending emails, and using functionalities like alarm clocks and notepads. Notably, they also exhibited a significant reduction in smartphone usage after work hours.

The correlation and regression analysis revealed a negative correlation of varying strengths between smartphone and app usage and nocturnal smartphone inactivity. Specifically, participants who frequently used social media apps exhibited higher smartphone usage at night. In contrast, this pattern was reversed among participants with a doctoral degree. They predominantly used their smartphones for gathering information and reducing their nighttime usage. As a result, the more time dedicated to acquiring information throughout the day, the longer the duration they tended to refrain from using their smartphones at night.

In this study, nocturnal smartphone inactivity was used as an indirect indicator of sleep behavior. Nocturnal smartphone inactivity exceeding 6 hours was used as a threshold to assess whether participants achieved sufficient sleep duration. The results showed that over 92% (n=988) of participants exhibited smartphone inactivity for more than 6 hours at nighttime, demonstrating a high degree of consistency in this behavior. Additionally, for some participants, smartphone inactivity consistently occurred between 22:00 and 06:00, aligning closely with the National Sleep Foundation’s recommendation of 7-9 hours of sleep per night for adults [34].

Numerous studies indicate that women tend to have a higher reliance on cell phones compared to men, a trend driven by societal pressures and safety concerns [35,36]. Women predominantly used their smartphones for socializing, often dedicating more time to social media and shopping apps, activities that offered them pleasure and relaxation [37]. In contrast, men showed a preference for gaming and watching videos [38]. Research analysis has indicated a clear trend: the total time spent on smartphone usage and the dependence on it tend to decrease with age. Individuals younger than 20 years, especially teenagers, emerge as the most frequent users [39,40]. These young people predominantly engage with their smartphones for communication, socializing, gaming, watching videos, and listening to music [41,42]. In contrast, older adults, facing relatively lower social costs and pressures, show less frequent and shorter durations of smartphone usage, primarily focusing on reading news, using the phone as a clock for reminders, and communicating with peers in a traditional phone manner [41]. Notably, a significant issue among many young people is the continuous nighttime use of smartphones, which leads to sleep disturbances. Furthermore, it has been observed that the earlier the age at which individuals start using smartphones, the greater their dependence on these devices tends to be [43]. Some research indicates that students from families with higher cultural and economic statuses tend to have an increased dependency on smartphones [44]. This trend also correlates with the educational levels of the parents; children of more educated parents generally exhibit a healthier relationship with smartphone usage [45]. Individuals with academic accomplishments often rely on smartphones as a primary tool for accessing valuable information. Specifically in India, research shows that an impressive 90% of medical students consider smartphones to be extremely beneficial for accessing educational content, listening to lectures, and taking notes [46].

In our study, we discovered a negative correlation between smartphone usage and nocturnal smartphone inactivity. Particularly, participants who frequently used social media apps were found to engage with their phones more extensively at night. Social media facilitates self-exploration and emotional expression while serving as a pivotal platform for medical communication, education, and disaster response [47,48]. Given these capabilities, it is clear that social media has emerged as the most favored smartphone app. Excessive engagement with social media has been associated with significant psychological risks, including heightened anxiety, increased vulnerability to depression, and diminished overall well-being [49]. Moreover, engaging with social media during nighttime can significantly impair sleep quality and duration [50,51]. Numerous research findings indicate that late-night smartphone use can lead to sleep disturbances, resulting in diminished sleep quality and daytime fatigue [52]. This adverse effect is possibly due to presleep smartphone usage, which can reduce the latency of rapid eye movement sleep, elevate electroencephalogram spectrum power, delay the onset of melatonin secretion, and potentially cause mood fluctuations in users [19,53].

This study investigates the intricacies of smartphone usage patterns, emphasizing the profound correlations between variables such as gender, age, and education level on smartphone usage. The results underscore a trend: the escalation in smartphone use perpetuates habitual behaviors, particularly nocturnal usage, potentially disrupts users’ sleep patterns. Nonetheless, amid the digital era, prudent smartphone usage holds promise in significantly enhancing life satisfaction across diverse demographics, including older adult populations [54]. Thus, a thorough exploration of mobile phone usage patterns and the identification of their determinants are crucial for fostering a balanced and health-conscious engagement with these technological tools.

### Strengths and Limitations

This study has several limitations that may affect the interpretation and generalizability of its findings.

First, the study lacks direct physiological data related to sleep patterns and instead relies on nocturnal smartphone inactivity as a proxy for sleep duration. While this proxy provides a reasonable approximation of an individual's sleep window, it may be influenced by several confounding factors, including nonsleep behaviors (eg, reading and chatting with friends), interrupted sleep (eg, waking up and briefly using the smartphone), and sleep behaviors (eg, falling asleep while their phone continues to play music or videos). These potential discrepancies between smartphone inactivity and actual sleep behavior could introduce bias into the analysis.

Second, the study encountered limitations in participant demographic data collection. A substantial proportion of respondents failed to provide complete demographic information during the questionnaire phase, resulting in a reduced response rate and limiting the ability to analyze associations between specific demographic characteristics and smartphone usage behaviors. Furthermore, although participants were categorized by gender, age, education level, and employment status, the uneven sample distribution across categories may have led to statistical imbalances, thereby affecting the robustness and stability of results in certain subgroups. Additionally, as the data sample was primarily drawn from participants in Germany, the generalizability of the findings to other cultural or regional contexts remains limited.

Third, despite the study’s year-long design (365 days), the actual data recording period varied significantly among participants, with some contributing data for only a few days. This limited recording duration reduces the ability to capture long-term smartphone usage patterns and behaviors, which in turn may compromise the representativeness of the findings and hinder the identification of sustained behavioral trends over time.

To enhance the reliability and generalizability of future research on the relationship between smartphone use and sleep, several improvements are recommended. First, incorporating multidimensional data validation is crucial. Objective data from wearable devices, such as smartwatches or fitness trackers, can provide precise measurements of key sleep metrics, including total sleep duration, sleep latency, wake-up times, and sleep quality (eg, proportion of deep sleep). In addition, standardized subjective sleep reports, such as the Pittsburgh Sleep Quality Index and Epworth Sleepiness Scale, can capture participants’ self-reported sleep quality and patterns, adding valuable context to the analysis. These combined data sources will improve the accuracy of the proxy indicator of nocturnal smartphone inactivity while enhancing the scientific rigor of future studies. Second, ensuring sample diversity and representativeness is essential to generalize findings to broader populations. This includes recruiting participants with diverse demographic characteristics such as age, gender, education level, and cultural background. Comprehensive questionnaires should also address potential correlations in smartphone usage, such as socioeconomic status, work environment, and psychological stress, to uncover complex behavioral patterns and mechanisms. Stratified sampling or targeted recruitment strategies can help balance subgroup distributions, minimizing statistical bias caused by uneven sample sizes. Lastly, optimizing data collection processes will reduce omissions and improve consistency. This could involve developing reminder functions within smartphone apps or wearable devices to prompt participants to record sleep data or complete questionnaires regularly. Furthermore, promoting sleep health awareness during the study could enhance participants’ engagement and the quality of the data collected. Together, these methodological enhancements will provide a more comprehensive and accurate understanding of the complex relationship between smartphone use and sleep behavior while ensuring the findings are robust, representative, and actionable.

### Practical Advice

Based on the results, we can recommend measures to curb excessive smartphone and social media usage. Such interventions include advocating for periodic digital detoxes; implementing smartphone usage reminders and constraints; using apps to oversee and control screen time; minimizing unnecessary notifications from news feeds, game updates, and shopping alerts; and developing educational programs to deepen users’ understanding of smartphones and raise awareness about the potential risks associated with their use [55,56]. Additionally, it is suggested that app developers consider tailoring smartphone apps to meet the unique emotional and physiological needs of older adults, thereby enhancing their digital satisfaction and online well-being [57,58].

### Conclusions

This study offers valuable insights into smartphone usage behaviors, enhancing our understanding of the nuanced relationship between smartphone usage and sleep. The findings establish a robust basis for developing targeted health interventions tailored to diverse demographic groups. Moreover, this research enhances our comprehension of how various population segments engage with smartphones and the potential ramifications of these behaviors on their daily lives.

## Appendix

Multimedia Appendix 1 Statistical analysis of the daily duration of smartphone app usage across various groups.

Multimedia Appendix 2 Statistical analysis of the proportion of daily usage of smartphone apps across various groups.

Multimedia Appendix 3 Statistical analysis of the daily duration of smartphone usage across various groups.

Multimedia Appendix 4 Dunn test of differences in the daily duration of smartphone app usage across various groups.

Multimedia Appendix 5 Dunn test of differences in the proportion of daily usage of smartphone apps across various groups.

Multimedia Appendix 6 Dunn test of differences in the daily duration of smartphone usage across various groups.

Multimedia Appendix 7 Statistical Analysis of Nocturnal Smartphone Inactivity Data Across Various Groups.

Multimedia Appendix 8 Dunn test of differences in nocturnal smartphone inactivity duration on days exceeding 6 hours.

Multimedia Appendix 9 Dunn test of differences in the proportion of days with >6 hours of nocturnal smartphone inactivity.

Multimedia Appendix 10 Correlation and Regression Heatmap: Linear Analysis of Daily Application Duration (Type A-E) and Daily Smartphone Usage in Relation to Nocturnal Inactivity Duration across Demographic Groups.

Multimedia Appendix 11 Correlation and regression analysis: comparing daily duration between smartphone app usage (types A, B, and C) and nocturnal smartphone inactivity.

Multimedia Appendix 12 Correlation and regression analysis: comparing daily duration between smartphone app usage (type D, E, and unknown) and nocturnal smartphone inactivity.

Multimedia Appendix 13 Correlation and regression analysis: comparing daily duration between smartphone usage and nocturnal smartphone inactivity.

## Abbreviations

STROBE: Strengthening the Reporting of Observational Studies in Epidemiology

## Data Availability

The datasets generated and analyzed during this study were collected and anonymized using the Murmuras app. Due to ethical and privacy considerations, the raw data are not publicly available. However, the data are available from the corresponding author on reasonable request.

## References

[1] (2024). Number of smartphone mobile network subscriptions worldwide from 2016 to 2023, with forecasts from 2023 to 2028. Statista.

[2] Chan M (2018). Mobile-mediated multimodal communications, relationship quality and subjective well-being: an analysis of smartphone use from a life course perspective. Comput Hum Behav.

[3] Oviedo-Trespalacios O, Nandavar S, Newton JDA, Demant D, Phillips JG (2019). Problematic use of mobile phones in Australia…is it getting worse?. Front Psychiatry.

[4] (2022). mHealth: use of appropriate digital technologies for public health: report by the Director-General. World Health Organization.

[5] Mekonnen ZA, Tilahun B, Alemu K, Were M (2019). Effect of mobile phone text message reminders on improving completeness and timeliness of routine childhood vaccinations in North-West, Ethiopia: a study protocol for randomised controlled trial. BMJ Open.

[6] Robbins R, Weaver MD, Quan SF, Sullivan JP, Cohen-Zion M, Glasner L, Qadri S, Czeisler CA, Barger LK (2022). A clinical trial to evaluate the dayzz smartphone app on employee sleep, health, and productivity at a large US employer. PLoS One.

[7] Al-Hadidi F, Bsisu I, AlRyalat SA, Al-Zu'bi B, Bsisu R, Hamdan M, Kanaan T, Yasin M, Samarah O (2019). Association between mobile phone use and neck pain in university students: a cross-sectional study using numeric rating scale for evaluation of neck pain. PLoS One.

[8] Elhai JD, Dvorak RD, Levine JC, Hall BJ (2017). Problematic smartphone use: a conceptual overview and systematic review of relations with anxiety and depression psychopathology. J Affect Disord.

[9] Jung SI, Lee NK, Kang KW, Kim K, Lee DY (2016). The effect of smartphone usage time on posture and respiratory function. J Phys Ther Sci.

[10] Lee HS, Park SW, Heo H (2016). Acute acquired comitant esotropia related to excessive Smartphone use. BMC Ophthalmol.

[11] Sohn SY, Rees P, Wildridge B, Kalk NJ, Carter B (2019). Prevalence of problematic smartphone usage and associated mental health outcomes amongst children and young people: a systematic review, meta-analysis and GRADE of the evidence. BMC Psychiatry.

[12] Kwon M, Lee JY, Won WY, Park JW, Min JA, Hahn C, Gu X, Choi JH, Kim DJ (2013). Development and validation of a smartphone addiction scale (SAS). PLoS One.

[13] King A, Valença A, Silva A, Baczynski T, Carvalho M, Nardi A (2013). Nomophobia: dependency on virtual environments or social phobia?. Comput Hum Behav.

[14] van Velthoven MH, Powell J, Powell G (2018). Problematic smartphone use: digital approaches to an emerging public health problem. Digit Health.

[15] Chatterjee S, Kar SK (2021). Smartphone addiction and quality of sleep among indian medical students. Psychiatry.

[16] Kaya F, Bostanci Daştan N, Durar E (2021). Smart phone usage, sleep quality and depression in university students. Int J Soc Psychiatry.

[17] Sohn SY, Krasnoff L, Rees P, Kalk NJ, Carter B (2021). The association between smartphone addiction and sleep: a UK cross-sectional study of young adults. Front Psychiatry.

[18] Lanaj K, Johnson RE, Barnes CM (2014). Beginning the workday yet already depleted? Consequences of late-night smartphone use and sleep. Organ Behav Hum Decis Process.

[19] Wood AW, Loughran SP, Stough C (2006). Does evening exposure to mobile phone radiation affect subsequent melatonin production?. Int J Radiat Biol.

[20] Browman CP, Tepas DI (1976). The effects of presleep activity on all-night sleep. Psychophysiology.

[21] Harvey AG, Greenall E (2003). Catastrophic worry in primary insomnia. J Behav Ther Exp Psychiatry.

[22] Matricciani L, Bin YS, Lallukka T, Kronholm E, Dumuid D, Paquet C, Olds T (2017). Past, present, and future: trends in sleep duration and implications for public health. Sleep Health.

[23] Paruthi S, Brooks LJ, D'Ambrosio C, Hall WA, Kotagal S, Lloyd RM, Malow BA, Maski K, Nichols C, Quan SF, Rosen CL, Troester MM, Wise MS (2016). Recommended amount of sleep for pediatric populations: a consensus statement of the American academy of sleep medicine. J Clin Sleep Med.

[24] Reutrakul S, Anothaisintawee T, Herring SJ, Balserak BI, Marc I, Thakkinstian A (2018). Short sleep duration and hyperglycemia in pregnancy: aggregate and individual patient data meta-analysis. Sleep Med Rev.

[25] Kim CE, Shin S, Lee H, Lim J, Lee J, Shin A, Kang D (2018). Association between sleep duration and metabolic syndrome: a cross-sectional study. BMC Public Health.

[26] Ciman M, Wac K (2019). Smartphones as sleep duration sensors: validation of the iSenseSleep algorithm. JMIR Mhealth Uhealth.

[27] Heimlich R (2010). Do you sleep with your cell phone?. Pew Research Center.

[28] Borger JN, Huber R, Ghosh A (2019). Capturing sleep-wake cycles by using day-to-day smartphone touchscreen interactions. NPJ Digit Med.

[29] Cuttone A, Bækgaard P, Sekara V, Jonsson H, Larsen JE, Lehmann S (2017). SensibleSleep: a Bayesian model for learning sleep patterns from smartphone events. PLoS One.

[30] Blackwelder A, Hoskins M, Huber L (2021). Effect of inadequate sleep on frequent mental distress. Prev Chronic Dis.

[31] Andone I, Blaszkiewicz K, Eibes M, Trendafilov B, Montag C, Markowetz A (2016). Menthal: quantifying smartphone usage.

[32] Arbeitskreis Medizinischer Ethik-Kommissionen.

[33] Shen Y Mobile phone usage and its influencing [PhD thesis]. Zhejiang University.

[34] Hirshkowitz M, Whiton K, Albert SM, Alessi C, Bruni O, DonCarlos L, Hazen N, Herman J, Katz ES, Kheirandish-Gozal L, Neubauer DN, O'Donnell AE, Ohayon M, Peever J, Rawding R, Sachdeva RC, Setters B, Vitiello MV, Ware JC, Adams Hillard PJ (2015). National sleep foundation's sleep time duration recommendations: methodology and results summary. Sleep Health.

[35] Lee KE, Kim S, Ha T, Yoo Y, Han J, Jung J, Jang J (2016). Dependency on smartphone use and its association with anxiety in Korea. Public Health Rep.

[36] Albursan IS, Al Qudah MF, Al-Barashdi HS, Bakhiet SF, Darandari E, Al-Asqah SS, Hammad HI, Al-Khadher MM, Qara S, Al-Mutairy SH, Albursan HI (2022). Smartphone addiction among university students in light of the COVID-19 pandemic: prevalence, relationship to academic procrastination, quality of life, gender and educational stage. Int J Environ Res Public Health.

[37] Mustafaoglu R, Yasaci Z, Zirek E, Griffiths MD, Ozdincler AR (2021). The relationship between smartphone addiction and musculoskeletal pain prevalence among young population: a cross-sectional study. Korean J Pain.

[38] Chen B, Liu F, Ding S, Ying X, Wang L, Wen Y (2017). Gender differences in factors associated with smartphone addiction: a cross-sectional study among medical college students. BMC Psychiatry.

[39] Horwood S, Anglim J, Mallawaarachchi SR (2021). Problematic smartphone use in a large nationally representative sample: age, reporting biases, and technology concerns. Comput Hum Behav.

[40] de Nadai M, Cardoso A, Lima A, Lepri B, Oliver N (2019). Strategies and limitations in app usage and human mobility. Sci Rep.

[41] Andone I, Blaszkiewicz K, Eibes M, Trendafilov B, Montag C, Markowetz A (2016). How age and gender affect smartphone usage.

[42] Hassanzadeh R, Rezaei A (2011). Effect of sex, course and age on SMS addiction in students. Middle-East J Sci Res.

[43] Sahin S, Ozdemir K, Unsal A, Temiz N (2013). Evaluation of mobile phone addiction level and sleep quality in university students. Pak J Med Sci.

[44] Mazaheri MA, Najarkolaei FR (2014). Cell phone and internet addiction among students in Isfahan University of Medical Sciences (Iran). Journal of Health Policy and Sustainable Health.

[45] López-Fernández O, Honrubia-Serrano ML, Freixa-Blanxart M (2012). Adaptación española del “Mobile Phone Problem Use Scale” para población adolescente. Adicciones.

[46] Latif MZ, Hussain I, Saeed R, Qureshi MA, Maqsood U (2019). Use of smart phones and social media in medical education: trends, advantages, challenges and barriers. Acta Inform Med.

[47] Gerwin RL, Kaliebe K, Daigle M (2018). The interplay between digital media use and development. Child Adolesc Psychiatr Clin N Am.

[48] Jamal A, Temsah M, Khan SA, Al-Eyadhy A, Koppel C, Chiang MF (2016). Mobile phone use among medical residents: a cross-sectional multicenter survey in Saudi Arabia. JMIR Mhealth Uhealth.

[49] Ahmed O, Walsh EI, Dawel A, Alateeq K, Espinoza Oyarce DA, Cherbuin N (2024). Social media use, mental health and sleep: a systematic review with meta-analyses. J Affect Disord.

[50] Sancho-Domingo C, Garmy P, Norell A (2024). Nighttime texting on social media, sleep parameters, and adolescent sadness: a mediation analysis. Behav Sleep Med.

[51] Meyerson WU, Fineberg SK, Andrade FC, Corlett P, Gerstein MB, Hoyle RH (2023). The association between evening social media use and delayed sleep may be causal: suggestive evidence from 120 million reddit timestamps. Sleep Med.

[52] Matar Boumosleh J, Jaalouk D (2017). Depression, anxiety, and smartphone addiction in university students - a cross sectional study. PLoS One.

[53] Gregory AM, Willis TA, Wiggs L, Harvey AG, STEPS Team (2008). Presleep arousal and sleep disturbances in children. Sleep.

[54] Rosenberg D, Taipale S (2022). Social and satisfied? Social uses of mobile phone and subjective wellbeing in later life. Hum Technol.

[55] Wei X, Zhou H, Zheng Q, Ren L, Chen N, Wang P, Liu C (2025). Longitudinal interactions between problematic internet gaming and symptoms of depression among university students: differentiating anhedonia and depressed mood. Addict Behav.

[56] Dissing AS, Andersen TO, Nørup LN, Clark A, Nejsum M, Rod NH (2021). Daytime and nighttime smartphone use: a study of associations between multidimensional smartphone behaviours and sleep among 24,856 Danish adults. J Sleep Res.

[57] Meade ME, Chang M, Savel K, Hong B, Martin CB, Barense MD (2024). Unique events improve episodic richness, enhance mood, and alter the perception of time during isolation. Sci Rep.

[58] Wang X, Zhao Y (2024). Does smartphone use affect attitudes toward aging among older adults in rural areas? An empirical analysis using data from the Chinese longitudinal aging social survey. Behav Sci (Basel).

