# Supplementary material for: Smartphone Usage Patterns and Sleep Behavior in Demographic Groups: Retrospective Observational Study
Source: J Med Internet Res. 2025 Jul 3;27:e60423. doi: 10.2196/60423 (PMC12271961; doi:10.2196/60423)
Supplement: Multimedia Appendix 1 [file jmir_v27i1e60423_app1.docx]

Multimedia Appendix 1. Statistical Analysis of Daily Duration of Smartphone Application Usage Across Various Groups (unit: h)

| Group Category | | Type A | | Type B | | Type C | | Type D | | Type E | | Type Unknown | |
| --- | --- | --- | --- | --- | --- | --- | --- | --- | --- | --- | --- | --- | --- |
|  |  | Median [Q1, Q3] (h) | *P*-value | Median [Q1, Q3] (h) | *P*-value | Median [Q1, Q3] (h) | *P*-value | Median [Q1, Q3] (h) | *P*-value | Median [Q1, Q3] (h) | *P*-value | Median [Q1, Q3] (h) | *P*-value |
| **Gender (n=1074)** | |  |  |  |  |  |  |  |  |  |  |  |  |
|  | Male (n=350) | 0.54 [0.27, 0.83] | .78 | 0.75 [0.28, 1.51] | .32 | 0.18[0.09, 0.32] | < .001 | 1.13[0.53, 1.76] | < .001 | 0.06[0.03, 0.12] | < .001 | 0.03[0.01, 0.08] | .02 |
|  | Female (n=724) | 0.52[0.32, 0.82] |  | 0.68 [0.23, 1.46] |  | 0.22[0.13, 0.37] |  | 1.45[0.95, 2.14] |  | 0.09[0.04, 0.17] |  | 0.04[0.02, 0.07] |  |
| **Age (n=1074)** | |  |  |  |  |  |  |  |  |  |  |  |  |
|  | Less than 18 years (n=8) | 0.32[0.21, 0.46] | .23 | 1.42 [0.35, 1.94] | < .001 | 0.20[0.15, 0.34] | .001 | 0.83[0.20, 1.80] | < .001 | 0.08[0.05, 0.16] | .15 | 0.03[0.02, 0.08] | .15 |
|  | 18 years or older < 35 years (n=895) | 0.54[0.32, 0.82] |  | 0.80 [0.29, 1.56] |  | 0.16[0.14, 0.31] |  | 1.42[0.85, 2.06] |  | 0.07[0.04, 0.16] |  | 0.03[0.01, 0.07] |  |
|  | 35 years or older < 60 years (n=161) | 0.48[0.28, 0.88] |  | 0.36 [0.13, 1.14] |  | 0.28[0.13, 0.50] |  | 1.15[0.70, 1.78] |  | 0.09[0.05, 0.16] |  | 0.04[0.02, 0.09] |  |
|  | 60 years or older (n=10) | 0.52[0.22, 0.74] |  | 0.15 [0.08, 0.84] |  | 0.16[0.14, 0.31] |  | 0.47[0.42, 0.82] |  | 0.06[0.05, 0.09] |  | 0.03[0.03, 0.07] |  |
| **Highest degree (n=1059)** | |  |  |  |  |  |  |  |  |  |  |  |  |
|  | Doctorate (n=11) | 0.43[0.18, 0.67] | < .001 | 0.41 [0.14, 1.14] | < .001 | 0.16[0.12, 0.25] | .70 | 1.25[0.76, 1.41] | < .001 | 0.07[0.02, 0.13] | .75 | 0.03[0.02, 0.07] | .96 |
|  | Master’s degree (n=116) | 0.55[0.33, 0.95] |  | 0.26 [0.09, 0.75] |  | 0.21[0.12, 0.38] |  | 0.91[0.48, 1.59] |  | 0.07[0.02, 0.13] |  | 0.04[0.02, 0.07] |  |
|  | Bachelor’s degree (n=189) | 0.59[0.31, 1.07] |  | 0.65 [0.20, 1.26] |  | 0.20[0.13, 0.34] |  | 1.25[0.84, 1.84] |  | 0.07[0.02, 0.15] |  | 0.03[0.01, 0.07] |  |
|  | Secondary education (n=96) | 0.43[0.18, 0.66] |  | 0.40 [0.13, 1.69] |  | 0.23[0.12, 0.39] |  | 1.23[0.53, 1.77] |  | 0.07[0.03, 0.14] |  | 0.03[0.02, 0.07] |  |
|  | High school degree or equivalent (n=637) | 0.53[0.33, 0.79] |  | 0.79 [0.28, 1.64] |  | 0.21[0.12, 0.34] |  | 1.44[0.83, 2.07] |  | 0.06[0.03, 0.14] |  | 0.03[0.01, 0.07] |  |
|  | No formal qualification (n=10) | 0.25[0.17, 0.53] |  | 0.80 [0.18, 1.49] |  | 0.23[0.17, 0.31] |  | 1.18[0.44, 3.03] |  | 0.03[0.000, 0.09] |  | 0.05[0.02, 0.07] |  |
| **Employment status (n=1043)** | |  |  |  |  |  |  |  |  |  |  |  |  |
|  | In education (n=535) | 0.54[0.33, 0.86] | .42 | 0.90 [0.28, 1.75] | < .001 | 0.20[0.12, 0.33] | .26 | 1.44[0.84, 2.07] | < .001 | 0.06[0.02, 0.12] | .003 | 0.03[0.01, 0.06] | .61 |
|  | Unemployed job-seeking (n=20) | 0.51[0.34, 1.05] |  | 1.13 [0.44, 2.97] |  | 0.29[0.19, 0.51] |  | 1.32[0.68, 2.55] |  | 0.04[0.03, 0.07] |  | 0.02[0.01, 0.07] |  |
|  | Part-time (n=149) | 0.53[0.29, 0.86] |  | 0.45 [0.15, 1.00] |  | 0.23[0.11, 0.36] |  | 1.11[0.63, 1.71] |  | 0.07[0.02, 0.17] |  | 0.03[0.02, 0.07] |  |
|  | Full-time (n=267) | 0.54[0.30, 0.81] |  | 0.46 [0.19, 1.28] |  | 0.21[0.13, 0.36] |  | 1.29[0.75, 1.89] |  | 0.08[0.03, 0.16] |  | 0.04[0.02, 0.07] |  |
|  | Self-employed (n=41) | 0.41[0.26, 0.75] |  | 0.27 [0.05, 0.54] |  | 0.20[0.11, 0.35] |  | 1.13[0.44, 2.13] |  | 0.04[0.001, 0.10] |  | 0.05[0.01, 0.11] |  |
|  | Homemaker (n=14) | 0.47[0.38, 0.66] |  | 0.69 [0.19, 1.47] |  | 0.18[0.13, 0.30] |  | 1.53[1.09, 3.11] |  | 0.12[0.07, 0.24] |  | 0.04[0.01, 0.11] |  |
|  | Retired (n=17) | 0.40[0.20, 0.53] |  | 0.14 [0.06, 0.24] |  | 0.14[0.12, 0.30] |  | 0.46[0.12, 1.25] |  | 0.05[0.001, 0.08] |  | 0.03[0.02, 0.07] |  |
| **Smartphone use type (n=1074)** | |  |  |  |  |  |  |  |  |  |  |  |  |
|  | Both equally (n=139) | 0.54[0.27, 0.84] | < .001 | 0.56 [0.12, 1.45] | < .001 | 0.20[0.12, 0.34] | .001 | 1.18[0.48, 1.87] | < .001 | 0.06[0.02, 0.14] | .007 | 0.03[0.01, 0.07] | .03 |
|  | Mainly private (n=390) | 0.58[0.36, 0.88] |  | 0.69 [0.22, 1.51] |  | 0.22[0.13, 0.36] |  | 1.35[0.81, 2.09] |  | 0.06[0.03, 0.13] |  | 0.04[0.02, 0.08] |  |
|  | Mainly work (n=14) | 0.13[0.07, 0.47] |  | 0.03 [0.00, 0.10] |  | 0.08[0.04, 0.16] |  | 0.14[0.03, 1.07] |  | 0.01[0.000, 0.05] |  | 0.01[0.004, 0.05] |  |
|  | Private only (n=524) | 0.50[0.30, 0.78] |  | 0.65 [0.22, 1.44] |  | 0.20[0.12, 0.35] |  | 1.36[0.81, 2.00] |  | 0.07[0.03, 0.15] |  | 0.03[0.01, 0.06] |  |
|  | Work only (n=7) | 0.05[0.05, 0.16] |  | 0.01 [0.003, 0.18] ​ |  | 0.03[0.02, 0.10] |  | 0.05[0.0008, 0.26] |  | 0.000[0.000, 0.09] |  | 0.01[0.003, 0.02] |  |

Note: Daily duration of smartphone application usage are represented by median values with the first (Q1) and third (Q3) quartiles.
