# Supplementary material for: Smartphone Usage Patterns and Sleep Behavior in Demographic Groups: Retrospective Observational Study
Source: J Med Internet Res. 2025 Jul 3;27:e60423. doi: 10.2196/60423 (PMC12271961; doi:10.2196/60423)
Supplement: Multimedia Appendix 2 [file jmir_v27i1e60423_app2.docx]

Multimedia Appendix 2. Statistical Analysis of Proportion of Daily Usage of Smartphone Application Across Various Groups (unit: %)

| Group Category | | Type A | | Type B | | Type C | | Type D | | Type E | | Type Unknown | |
| --- | --- | --- | --- | --- | --- | --- | --- | --- | --- | --- | --- | --- | --- |
|  |  | Median [Q1, Q3] (%) | *P*-value | Median [Q1, Q3] (%) | *P*-value | Median [Q1, Q3] (%) | *P*-value | Median [Q1, Q3] (%) | *P*-value | Median [Q1, Q3] (%) | *P*-value | Median [Q1, Q3] (%) | *P*-value |
| **Gender (n=1074)** | |  |  |  |  |  |  |  |  |  |  |  |  |
|  | Male (n=350) | 13.25[8.81, 20.03] | < .001 | 6.87[4.20, 11.05] | .45 | 40.46[34.67, 45.59] | .29 | 30.59[21.59, 36.62] | < .001 | 0.69[0.07, 2.24] | .006 | 2.55[1.25, 6.37] | .69 |
|  | Female (n=724) | 11.87 [8.22, 16.20] |  | 6.72[4.22, 9.80] |  | 40.97[36.08, 45.34] |  | 32.77[26.97, 38.27] |  | 0.93[0.30, 2.34] |  | 2.63[1.56, 4.47] |  |
| **Age (n=1074)** | |  |  |  |  |  |  |  |  |  |  |  |  |
|  | Less than 18 years (n=8) | 12.20 [7.95, 25.98] | .004 | 7.03[1.97, 11.72] | < .001 | 40.23[32.89, 47.74] | .94 | 21.57[6.82, 37.99] | .10 | 0.18[0.00, 1.21] | .07 | 8.42[4.08, 12.05] | < .001 |
|  | 18 years or older < 35 years (n=895) | 11.98[8.32, 16.82] |  | 7.01[4.37, 10.56] |  | 40.84[35.73, 45.57] |  | 32.36[26.14, 38.12] |  | 0.83[0.23, 2.10] |  | 2.50[1.42, 4.52] |  |
|  | 35 years or older < 60 years (n=161) | 14.86[9.43, 21.00] |  | 5.68[3.52, 8.12] |  | 40.49[35.72, 44.54] |  | 31.01[24.84, 36.08] |  | 1.16[0.27, 3.04] |  | 3.59[1.89, 6.71] |  |
|  | 60 years or older (n=10) | 19.25[8.25, 26.49] |  | 6.91 [5.60, 8.75] |  | 39.56 [34.53, 47.29] |  | 32.60[16.71, 36.48] |  | 1.25[0.16, 2.87] |  | 2.80[1.77, 6.29] |  |
| **Highest degree (n=1059)** | |  |  |  |  |  |  |  |  |  |  |  |  |
|  | Doctorate (n=11) | 12.39[11.03, 15.27] | < .001 | 6.03 [4.39, 11.89] | < .001 | 39.52[34.30, 40.49] | .12 | 33.02[26.11, 35.22] | .10 | 0.60[0.11, 1.92] | .10 | 4.72[2.71, 9.87] | .04 |
|  | Master’s degree (n=116) | 16.03[9.73, 23.27] |  | 5.67 [3.27, 7.90] |  | 40.78 [35.17, 45.48] |  | 29.64[20.10, 36.77] |  | 0.89[0.23 2.53] |  | 2.94[1.58, 5.19] |  |
|  | Bachelor’s degree (n=189) | 13.35[10.30, 19.36] |  | 6.52[4.07, 9.79] |  | 39.39 [35.02, 44.20] |  | 31.78[26.42, 37.25] |  | 0.89[0.29, 2.77] |  | 2.70[1.64, 4.78] |  |
|  | Secondary education (n=96) | 12.44[7.78, 17.86] |  | 6.31[3.21, 8.14] |  | 41.91 [36.45, 45.30] |  | 30.56[25.64, 36.41] |  | 1.24[0.22, 3.65] |  | 2.87[1.82, 5.82] |  |
|  | High school degree or equivalent (n=637) | 11.52[7.97, 15.92] |  | 7.30[4.65, 10.73] |  | 41.03[36.01, 45.80] |  | 32.67[26.17, 38.33] |  | 0.84[0.25, 1.99] |  | 2.46[1.36, 4.43] |  |
|  | No formal qualification (n=10) | 8.76[5.28, 13.21] |  | 5.44[2.10, 9.30] |  | 43.02 [37.92, 49.81] |  | 32.99[22.16, 44.16] |  | 0.17[0.00, 0.28] |  | 2.94[0.89, 7.49] |  |
| **Employment status (n=1043)** | |  |  |  |  |  |  |  |  |  |  |  |  |
|  | In education (n=535) | 11.51[8.12, 16.21] | .006 | 7.26[4.81, 10.89] | < .001 | 40.67 [35.76, 45.75] | .09 | 32.63[26.07, 38.26] | .52 | 0.71[0.23, 1.80] | < .001 | 2.37[1.30, 4.08] | < .001 |
|  | Unemployed job-seeking (n=20) | 11.23[9.18, 14.60] |  | 9.44[6.241, 14.55] |  | 41.13[37.60, 45.50] |  | 32.21[25.27, 37.75] |  | 0.29[0.11, 1.03] |  | 2.17[0.58, 3.33] |  |
|  | Part-time (n=149) | 14.03[9.58, 19.26] |  | 5.72[3.82, 8.58] |  | 40.70 [36.20, 45.31] |  | 32.05[25.01, 37.37] |  | 1.04[0.23, 3.46] |  | 2.90[1.78, 5.55] |  |
|  | Full-time (n=267) | 12.56[8.35, 17.54] |  | 6.39[3.98, 9.70] |  | 40.87[35.73, 44.98] |  | 31.12[26.16, 37.40] |  | 1.30[0.47, 3.00] |  | 2.82[1.70, 5.15] |  |
|  | Self-employed (n=41) | 15.30[8.56, 21.31] |  | 4.64[1.69, 7.30] |  | 40.47[35.80, 45.75] |  | 34.42[23.23, 40.12] |  | 0.60[0.01, 2.72] |  | 3.35[1.96, 7.41] |  |
|  | Homemaker (n=14) | 9.46[8.21, 16.34] |  | 5.95[3.38, 9.15] |  | 34.29[22.69, 38.83] |  | 31.65 [24.38, 38.45] |  | 2.70[1.05, 3.82] |  | 3.34[1.33, 5.31] |  |
|  | Retired (n=17) | 16.98[11.22, 26.41] |  | 6.51[4.07, 9.07] |  | 38.12[33.42, 47.44] |  | 29.48[19.50, 33.36] |  | 0.45[0.03, 1.89] |  | 4.93[2.40, 7.30] |  |
| **Smartphone use type(n=1074)** | |  |  |  |  |  |  |  |  |  |  |  |  |
|  | Both equally (n=139) | 13.37[9.13, 18.98] | .006 | 6.14[3.30, 9.07] | < .001 | 41.03[35.17, 45.02] | .82 | 32.44[21.05, 37.45] | < .001 | 0.74[0.23, 2.33] | .01 | 2.86[1.74, 6.99] | .002 |
|  | Mainly private (n=390) | 12.62[8.43, 17.12] |  | 6.88[4.73, 10.79] |  | 40.36[36.10, 45.43] |  | 32.60[26.56, 38.00] |  | 0.96[0.26, 2.36] |  | 2.62[1.46, 4.54] |  |
|  | Mainly work (n=14) | 16.61[11.12, 31.99] |  | 3.47[0.00, 5.02] |  | 41.96[37.63, 47.17] |  | 16.79[5.48, 27.91] |  | 0.14[0.00, 1.07] |  | 7.10[5.31, 11.75] |  |
|  | Private only (n=524) | 11.65[8.20, 16.62] |  | 7.04[4.34, 10.21] |  | 40.94[35.70, 45.36] |  | 32.02[26.20, 37.89] |  | 0.87[0.26, 2.26] |  | 2.49[1.40, 4.53] |  |
|  | Work only (n=7) | 31.76[19.86, 38.62] |  | 3.36[1.30, 6.41] |  | 42.08[40.35, 46.00] |  | 18.42 [2.70, 26.81] |  | 0.00[0.00, 0.10] |  | 8.00[4.01, 15.31] |  |

Note: Proportions of daily usage of smartphone application are represented by median values with the first (Q1) and third (Q3) quartiles.
