# Supplementary material for: Smartphone Usage Patterns and Sleep Behavior in Demographic Groups: Retrospective Observational Study
Source: J Med Internet Res. 2025 Jul 3;27:e60423. doi: 10.2196/60423 (PMC12271961; doi:10.2196/60423)
Supplement: Multimedia Appendix 3 [file jmir_v27i1e60423_app3.docx]

Multimedia Appendix 3. Statistical Analysis of Daily Duration of Smartphone Usage Across Various Groups

| Research object | | Total Participants in this research | | |
| --- | --- | --- | --- | --- |
| Group Category | | Number of Participants | Daily Duration of Smartphone Usage | |
|  |  |  | Median [Q1, Q3] (h) | *P*-value |
| **Gender** | |  |  |  |
|  | Male | 350 | 3.14 [1.72, 4.28] | < .001 |
|  | Female | 724 | 3.49 [2.36, 4.83] |  |
| **Age** | |  |  |  |
|  | Less than 18 years | 8 | 3.01 [1.21, 4.26] | .002 |
|  | 18 years or older < 35 years | 895 | 3.46 [2.31, 4.73] |  |
|  | 35 years or older < 60 years | 161 | 2.89 [1.76, 4.49] |  |
|  | 60 years or older | 10 | 1.85 [0.72, 2.39] |  |
| **Highest degree** | |  |  |  |
|  | Doctorate | 11 | 2.26 [1.61, 4.00] | < .001 |
|  | Master’s degree | 116 | 2.60 [1.76, 3.77] |  |
|  | Bachelor’s degree | 189 | 3.37 [2.24, 4.30] |  |
|  | Secondary education | 96 | 2.91 [1.58, 4.59] |  |
|  | High school degree or equivalent | 637 | 3.58 [2.41, 4.92] |  |
|  | No formal qualification | 10 | 4.03 [1.98, 5.83] |  |
| **Employment status** | |  |  |  |
|  | In education | 535 | 3.68 [2.46, 4.94] | < .001 |
|  | Unemployed job-seeking | 20 | 4.46 [3.02, 7.71] |  |
|  | Part-time | 149 | 2.85 [1.93, 4.06] |  |
|  | Full-time | 267 | 3.20 [2.16, 4.32] |  |
|  | Self-employed | 41 | 2.46 [1.32, 4.54] |  |
|  | Homemaker | 14 | 4.28 [3.43, 6.08] |  |
|  | Retired | 17 | 1.48 [0.64, 2.34] |  |
| **Smartphone use type** | |  |  |  |
|  | Both equally | 139 | 3.17 [1.87, 4.82] | < .001 |
|  | Mainly private | 390 | 3.52 [2.38, 4.81] |  |
|  | Mainly work | 14 | 0.43 [0.11, 2.00] |  |
|  | Private only | 524 | 3.35 [2.26, 4.61] |  |
|  | Work only | 7 | 0.17 [0.08, 0.69] |  |

Note: Daily duration of smartphone usage data are represented by median values with the first (Q1) and third (Q3) quartiles.
