# Supplementary material for: Smartphone Usage Patterns and Sleep Behavior in Demographic Groups: Retrospective Observational Study
Source: J Med Internet Res. 2025 Jul 3;27:e60423. doi: 10.2196/60423 (PMC12271961; doi:10.2196/60423)
Supplement: Multimedia Appendix 4 [file jmir_v27i1e60423_app4.docx]

Multimedia Appendix 4. Dunn's Test of Differences in Daily Duration of Smartphone Application Usage Across Various Groups

| Indicator | Group Category | Comparison | Z Value | *P* Value Uncorrected | *P* Value Before | *P* Value Adjusted |
| --- | --- | --- | --- | --- | --- | --- |
| **Type A** | | |  |  |  |  |
|  | **Highest degree** | |  |  |  |  |
|  |  | “Bachelor’s degree” - “Doctorate” | 1.42 | 0.078 | 0.078 | 1.0000 |
|  |  | “Bachelor’s degree” - “High school degree or equivalent” | 1.94 | 0.0261 | 0.0261 | 0.3913 |
|  |  | “Doctorate” - “High school degree or equivalent” | -0.92 | 0.1793 | 0.1793 | 1.0000 |
|  |  | “Bachelor’s degree” - “Master’s degree” | 0.11 | 0.4575 | 0.4575 | 1.0000 |
|  |  | “Doctorate” - “Master’s degree” | -1.35 | 0.0878 | 0.0878 | 1.0000 |
|  |  | “High school degree or equivalent” - “Master’s degree” | -1.47 | 0.071 | 0.071 | 1.0000 |
|  |  | “Bachelor’s degree” - “No formal qualification” | 2.60 | 0.0047 | 0.0047 | 0.0702 |
|  |  | “Doctorate” - “No formal qualification” | 0.92 | 0.178 | 0.178 | 1.0000 |
|  |  | “High school degree or equivalent” - “No formal qualification” | 2.14 | 0.0161 | 0.0161 | 0.2420 |
|  |  | “Master’s degree” - “No formal qualification” | 2.52 | 0.0059 | 0.0059 | 0.0880 |
|  |  | “Bachelor’s degree” - “Secondary education” | 3.87 | 0.0001 | 0.0001 | 0.0008 |
|  |  | “Doctorate” - “Secondary education” | 0.14 | 0.4435 | 0.4435 | 1.0000 |
|  |  | “High school degree or equivalent” - “Secondary education” | 2.96 | 0.0015 | 0.0015 | 0.0229 |
|  |  | “Master’s degree” - “Secondary education” | 3.43 | 0.0003 | 0.0003 | 0.0046 |
|  |  | “No formal qualification” - “Secondary education” | -1.08 | 0.1407 | 0.1407 | 1.0000 |
|  | **Smartphone use type** | |  |  |  |  |
|  |  | “Both equally” - “Mainly private” | -1.82 | 0.0344 | 0.0344 | 0.3438 |
|  |  | “Both equally” - “Mainly work” | 3.03 | 0.0012 | 0.0012 | 0.0122 |
|  |  | “Mainly private” - “Mainly work” | 3.78 | 0.0001 | 0.0001 | 0.0008 |
|  |  | “Both equally” - “Private only” | 0.25 | 0.4007 | 0.4007 | 1.0000 |
|  |  | “Mainly private” - “Private only” | 3.05 | 0.0012 | 0.0012 | 0.0116 |
|  |  | “Mainly work” - “Private only” | -3.05 | 0.0011 | 0.0011 | 0.0115 |
|  |  | “Both equally” - “Work only” | 2.74 | 0.0031 | 0.0031 | 0.0308 |
|  |  | “Mainly private” - “Work only” | 3.25 | 0.0006 | 0.0006 | 0.0057 |
|  |  | “Mainly work” - “Work only” | 0.46 | 0.3238 | 0.3238 | 1.0000 |
|  |  | “Private only” - “Work only” | 2.73 | 0.0032 | 0.0032 | 0.0320 |
| **Type B** | | |  |  |  |  |
|  | **Age** | |  |  |  |  |
|  |  | “Less than 18 years” - “60 years or older” | 1.24 | 0.1079 | 0.1079 | 0.6476 |
|  |  | “Less than 18 years” - “18 years or older < 35 years” | -0.21 | 0.4161 | 0.4161 | 1.0000 |
|  |  | “60 years or older” - “18 years or older < 35 years” | -2.08 | 0.0186 | 0.0186 | 0.1119 |
|  |  | “Less than 18 years” - “35 years or older < 60years” | 0.93 | 0.1768 | 0.1768 | 1.0000 |
|  |  | “60 years or older” - “35 years or older < 60years” | -0.77 | 0.2206 | 0.2206 | 1.0000 |
|  |  | “18 years or older < 35 years” - “35 years or older < 60years” | 4.80 | 0.0000 | 0.0000 | 0.0000 |
|  | **Highest degree** | |  |  |  |  |
|  |  | “Bachelor’s degree” - “Doctorate” | 0.64 | 0.2627 | 0.2627 | 1.0000 |
|  |  | “Bachelor’s degree” - “High school degree or equivalent” | -2.19 | 0.0141 | 0.0141 | 0.2117 |
|  |  | “Doctorate” - “High school degree or equivalent” | -1.25 | 0.1065 | 0.1065 | 1.0000 |
|  |  | “Bachelor’s degree” - “Master’s degree” | 4.06 | 0.0000 | 0.0000 | 0.0004 |
|  |  | “Doctorate” - “Master’s degree” | 0.89 | 0.1859 | 0.1859 | 1.0000 |
|  |  | “High school degree or equivalent” - “Master’s degree” | 6.54 | 0.0000 | 0.0000 | 0.0000 |
|  |  | “Bachelor’s degree” - “No formal qualification” | -0.08 | 0.4664 | 0.4664 | 1.0000 |
|  |  | “Doctorate” - “No formal qualification” | -0.51 | 0.3038 | 0.3038 | 1.0000 |
|  |  | “High school degree or equivalent” - “No formal qualification” | 0.48 | 0.3141 | 0.3141 | 1.0000 |
|  |  | “Master’s degree” - “No formal qualification” | -1.54 | 0.0623 | 0.0623 | 0.9350 |
|  |  | “Bachelor’s degree” - “Secondary education” | 0.64 | 0.2606 | 0.2606 | 1.0000 |
|  |  | “Doctorate” - “Secondary education” | -0.37 | 0.3571 | 0.3571 | 1.0000 |
|  |  | “High school degree or equivalent” - “Secondary education” | 2.39 | 0.0083 | 0.0083 | 0.1249 |
|  |  | “Master’s degree” - “Secondary education” | -2.89 | 0.0019 | 0.0019 | 0.0292 |
|  |  | “No formal qualification” - “Secondary education” | 0.32 | 0.3729 | 0.3729 | 1.0000 |
|  | **Employment status** | |  |  |  |  |
|  |  | “Full-time” - “Homemaker” | -0.24 | 0.4048 | 0.4048 | 1.0000 |
|  |  | “Full-time” - “In education” | -3.95 | 0.0000 | 0.0000 | 0.0008 |
|  |  | “Homemaker” - “In education” | -0.85 | 0.1977 | 0.1977 | 1.0000 |
|  |  | “Full-time” - “Part-time” | 1.30 | 0.0968 | 0.0968 | 1.0000 |
|  |  | “Homemaker” - “Part-time” | 0.71 | 0.2383 | 0.2383 | 1.0000 |
|  |  | “In education” - “Part-time” | 4.63 | 0.0000 | 0.0000 | 0.0000 |
|  |  | “Full-time” - “Retired” | 3.13 | 0.0009 | 0.0009 | 0.0183 |
|  |  | “Homemaker” - “Retired” | 2.35 | 0.0093 | 0.0093 | 0.1958 |
|  |  | “In education” - “Retired” | 4.38 | 0.0000 | 0.0000 | 0.0001 |
|  |  | “Part-time” - “Retired” | 2.54 | 0.0056 | 0.0056 | 0.1166 |
|  |  | “Full-time” - “Self-employed” | 3.08 | 0.0010 | 0.0010 | 0.0219 |
|  |  | “Homemaker” - “Self-employed” | 1.88 | 0.0300 | 0.0300 | 0.6295 |
|  |  | “In education” - “Self-employed” | 5.01 | 0.0000 | 0.0000 | 0.0000 |
|  |  | “Part-time” - “Self-employed” | 2.17 | 0.0149 | 0.0149 | 0.3122 |
|  |  | “Retired” - “Self-employed” | -0.92 | 0.1776 | 0.1776 | 1.0000 |
|  |  | “Full-time” - “Unemployed/job-seeking” | -2.212 | 0.0132 | 0.0132 | 0.2779 |
|  |  | “Homemaker” - “Unemployed/job-seeking” | -1.29 | 0.0991 | 0.0991 | 1.0000 |
|  |  | “In education” - “Unemployed/job-seeking” | -0.96 | 0.1689 | 0.1689 | 1.0000 |
|  |  | “Part-time” - “Unemployed/job-seeking” | -2.72 | 0.0033 | 0.0033 | 0.0688 |
|  |  | “Retired” - “Unemployed/job-seeking” | -3.93 | 0.0000 | 0.0000 | 0.0009 |
|  |  | “Self-employed” - “Unemployed/job-seeking” | -3.78 | 0.0001 | 0.0001 | 0.0017 |
|  | **Smartphone use type** | |  |  |  |  |
|  |  | “Both equally” - “Mainly private” | -1.52 | 0.0649 | 0.0649 | 0.6489 |
|  |  | “Both equally” - “Mainly work” | 4.56 | 0.0000 | 0.0000 | 0.0000 |
|  |  | “Mainly private” - “Mainly work” | 5.25 | 0.0000 | 0.0000 | 0.0000 |
|  |  | “Both equally” - “Private only” | -1.05 | 0.1467 | 0.1467 | 1.0000 |
|  |  | “Mainly private” - “Private only” | 0.74 | 0.2301 | 0.2301 | 1.0000 |
|  |  | “Mainly work” - “Private only” | -5.09 | 0.0000 | 0.0000 | 0.0000 |
|  |  | “Both equally” - “Work only” | 3.06 | 0.0011 | 0.0011 | 0.0109 |
|  |  | “Mainly private” - “Work only” | 3.50 | 0.0002 | 0.0002 | 0.0023 |
|  |  | “Mainly work” - “Work only” | -0.20 | 0.4205 | 0.4205 | 1.0000 |
|  |  | “Private only” - “Work only” | 3.38 | 0.0004 | 0.0004 | 0.0036 |
| **Type C** | | |  |  |  |  |
|  | **Gender** | |  |  |  |  |
|  |  | "Female" - "Male" | 3.53 | 0.0002 | 0.0002 | 0.0002 |
|  | **Age** | |  |  |  |  |
|  |  | “Less than 18 years” - “60 years or older” | 0.21 | 0.4169 | 0.4169 | 1.0000 |
|  |  | “Less than 18 years” - “18 years or older < 35 years” | 0.20 | 0.4225 | 0.4225 | 1.0000 |
|  |  | “60 years or older” - “18 years or older < 35 years” | -0.09 | 0.4622 | 0.4622 | 1.0000 |
|  |  | “Less than 18 years” - “35 years or older < 60years” | -0.75 | 0.2281 | 0.2281 | 1.0000 |
|  |  | “60 years or older” - “35 years or older < 60years” | -1.13 | 0.1285 | 0.1285 | 0.7708 |
|  |  | “18 years or older < 35 years” - “35 years or older < 60years” | -3.96 | 0.0000 | 0.0000 | 0.0002 |
|  | **Smartphone use type** | |  |  |  |  |
|  |  | “Both equally” - “Mainly private” | -0.85 | 0.1988 | 0.1988 | 1.0000 |
|  |  | “Both equally” - “Mainly work” | 2.37 | 0.0088 | 0.0088 | 0.0885 |
|  |  | “Mainly private” - “Mainly work” | 2.75 | 0.0030 | 0.0030 | 0.0296 |
|  |  | “Both equally” - “Private only” | -0.22 | 0.4117 | 0.4117 | 1.0000 |
|  |  | “Mainly private” - “Private only” | 0.93 | 0.1759 | 0.1759 | 1.0000 |
|  |  | “Mainly work” - “Private only” | -2.53 | 0.0056 | 0.0056 | 0.0563 |
|  |  | “Both equally” - “Work only” | 2.96 | 0.0015 | 0.0015 | 0.0153 |
|  |  | “Mainly private” - “Work only” | 3.23 | 0.0006 | 0.0006 | 0.0062 |
|  |  | “Mainly work” - “Work only” | 1.04 | 0.1487 | 0.1487 | 1.0000 |
|  |  | “Private only” - “Work only” | 3.07 | 0.0011 | 0.0011 | 0.0106 |
| **Type D** | | |  |  |  |  |
|  | **Gender** | |  |  |  |  |
|  |  | "Female" - "Male" | 6.74 | 0.0000 | 0.0000 | 0.0000 |
|  | **Age** | |  |  |  |  |
|  |  | “Less than 18 years” - “60 years or older” | 0.98 | 0.1626 | 0.1626 | 0.9758 |
|  |  | “Less than 18 years” - “18 years or older < 35 years” | -1.66 | 0.0485 | 0.0485 | 0.2909 |
|  |  | “60 years or older” - “18 years or older < 35 years” | -3.32 | 0.0004 | 0.0004 | 0.0027 |
|  |  | “Less than 18 years” - “35 years or older < 60years” | -1.11 | 0.1345 | 0.1345 | 0.8069 |
|  |  | “60 years or older” - “35 years or older < 60years” | -2.66 | 0.0039 | 0.0039 | 0.0234 |
|  |  | “18 years or older < 35 years” - “35 years or older < 60years” | 2.21 | 0.0136 | 0.0136 | 0.0818 |
|  | **Highest degree** | |  |  |  |  |
|  |  | “Bachelor’s degree” - “Doctorate” | 0.71 | 0.2376 | 0.2376 | 1.0000 |
|  |  | “Bachelor’s degree” - “High school degree or equivalent” | -1.52 | 0.0638 | 0.0638 | 0.9566 |
|  |  | “Doctorate” - “High school degree or equivalent” | -1.14 | 0.1265 | 0.1265 | 1.0000 |
|  |  | “Bachelor’s degree” - “Master’s degree” | 2.54 | 0.0056 | 0.0056 | 0.0842 |
|  |  | “Doctorate” - “Master’s degree” | 0.25 | 0.4028 | 0.4028 | 1.0000 |
|  |  | “High school degree or equivalent” - “Master’s degree” | 4.21 | 0.0000 | 0.0000 | 0.0002 |
|  |  | “Bachelor’s degree” - “No formal qualification” | -0.13 | 0.4476 | 0.4476 | 1.0000 |
|  |  | “Doctorate” - “No formal qualification” | -0.60 | 0.2727 | 0.2727 | 1.0000 |
|  |  | “High school degree or equivalent” - “No formal qualification” | 0.26 | 0.3967 | 0.3967 | 1.0000 |
|  |  | “Master’s degree” - “No formal qualification” | -1.04 | 0.1498 | 0.1498 | 1.0000 |
|  |  | “Bachelor’s degree” - “Secondary education” | 1.078 | 0.1402 | 0.1402 | 1.0000 |
|  |  | “Doctorate” - “Secondary education” | -0.27 | 0.3933 | 0.3933 | 1.0000 |
|  |  | “High school degree or equivalent” - “Secondary education” | 2.39 | 0.0085 | 0.0085 | 0.1269 |
|  |  | “Master’s degree” - “Secondary education” | -1.19 | 0.1176 | 0.1176 | 1.0000 |
|  |  | “No formal qualification” - “Secondary education” | 0.54 | 0.2961 | 0.2961 | 1.0000 |
|  | **Employment status** | |  |  |  |  |
|  |  | “Full-time” - “Homemaker” | -1.43 | 0.0757 | 0.0757 | 1.0000 |
|  |  | “Full-time” - “In education” | -1.65 | 0.0499 | 0.0499 | 1.0000 |
|  |  | “Homemaker” - “In education” | 1.00 | 0.1593 | 0.1593 | 1.0000 |
|  |  | “Full-time” - “Part-time” | 1.86 | 0.0313 | 0.0313 | 0.6582 |
|  |  | “Homemaker” - “Part-time” | 2.09 | 0.0184 | 0.0184 | 0.3865 |
|  |  | “In education” - “Part-time” | 3.39 | 0.0004 | 0.0004 | 0.0074 |
|  |  | “Full-time” - “Retired” | 2.97 | 0.0015 | 0.0015 | 0.0308 |
|  |  | “Homemaker” - “Retired” | 3.15 | 0.0008 | 0.0008 | 0.0171 |
|  |  | “In education” - “Retired” | 3.52 | 0.0002 | 0.0002 | 0.0045 |
|  |  | “Part-time” - “Retired” | 2.16 | 0.0153 | 0.0153 | 0.3210 |
|  |  | “Full-time” - “Self-employed” | 0.60 | 0.2753 | 0.2753 | 1.0000 |
|  |  | “Homemaker” - “Self-employed” | 1.59 | 0.0555 | 0.0555 | 1.0000 |
|  |  | “In education” - “Self-employed” | 1.38 | 0.0840 | 0.0840 | 1.0000 |
|  |  | “Part-time” - “Self-employed” | -0.51 | 0.3045 | 0.3045 | 1.0000 |
|  |  | “Retired” - “Self-employed” | -2.23 | 0.0128 | 0.0128 | 0.2690 |
|  |  | “Full-time” - “Unemployed/job-seeking” | -0.82 | 0.2049 | 0.2049 | 1.0000 |
|  |  | “Homemaker” - “Unemployed/job-seeking” | 0.58 | 0.2809 | 0.2809 | 1.0000 |
|  |  | “In education” - “Unemployed/job-seeking” | -0.30 | 0.3830 | 0.3830 | 1.0000 |
|  |  | “Part-time” - “Unemployed/job-seeking” | -1.60 | 0.0546 | 0.0546 | 1.0000 |
|  |  | “Retired” - “Unemployed/job-seeking” | -2.83 | 0.0023 | 0.0023 | 0.0482 |
|  |  | “Self-employed” - “Unemployed/job-seeking” | -1.07 | 0.1428 | 0.1428 | 1.0000 |
|  | **Smartphone use type** | |  |  |  |  |
|  |  | “Both equally” - “Mainly private” | -2.46 | 0.0069 | 0.0069 | 0.0686 |
|  |  | “Both equally” - “Mainly work” | 2.50 | 0.0062 | 0.0062 | 0.0624 |
|  |  | “Mainly private” - “Mainly work” | 3.47 | 0.0003 | 0.0003 | 0.0026 |
|  |  | “Both equally” - “Private only” | -2.21 | 0.0135 | 0.0135 | 0.1351 |
|  |  | “Mainly private” - “Private only” | 0.49 | 0.3136 | 0.3136 | 1.0000 |
|  |  | “Mainly work” - “Private only” | -3.37 | 0.0004 | 0.0004 | 0.0038 |
|  |  | “Both equally” - “Work only” | 3.40 | 0.0003 | 0.0003 | 0.0033 |
|  |  | “Mainly private” - “Work only” | 4.09 | 0.0000 | 0.0000 | 0.0002 |
|  |  | “Mainly work” - “Work only” | 1.33 | 0.0912 | 0.0912 | 0.9123 |
|  |  | “Private only” - “Work only” | 4.02 | 0.0000 | 0.0000 | 0.0003 |
| **Type E** | | |  |  |  |  |
|  | **Gender** | |  |  |  |  |
|  |  | "Female" - "Male" | 5.82 | 0.0000 | 0.0000 | 0.0000 |
|  | **Employment status** | |  |  |  |  |
|  |  | “Full-time” - “Homemaker” | -1.40 | 0.0812 | 0.0812 | 1.0000 |
|  |  | “Full-time” - “In education” | 3.00 | 0.0013 | 0.0013 | 0.0282 |
|  |  | “Homemaker” - “In education” | 2.25 | 0.0124 | 0.0124 | 0.2595 |
|  |  | “Full-time” - “Part-time” | 1.15 | 0.1256 | 0.1256 | 1.0000 |
|  |  | “Homemaker” - “Part-time” | 1.79 | 0.0367 | 0.0367 | 0.7708 |
|  |  | “In education” - “Part-time” | -1.16 | 0.1227 | 0.1227 | 1.0000 |
|  |  | “Full-time” - “Retired” | 2.08 | 0.0188 | 0.0188 | 0.3943 |
|  |  | “Homemaker” - “Retired” | 2.50 | 0.0062 | 0.0062 | 0.1293 |
|  |  | “In education” - “Retired” | 1.20 | 0.1153 | 0.1153 | 1.0000 |
|  |  | “Part-time” - “Retired” | 1.57 | 0.0578 | 0.0578 | 1.0000 |
|  |  | “Full-time” - “Self-employed” | 2.83 | 0.0023 | 0.0023 | 0.0493 |
|  |  | “Homemaker” - “Self-employed” | 2.77 | 0.0028 | 0.0028 | 0.0589 |
|  |  | “In education” - “Self-employed” | 1.54 | 0.0620 | 0.0620 | 1.0000 |
|  |  | “Part-time” - “Self-employed” | 2.02 | 0.0215 | 0.0215 | 0.4518 |
|  |  | “Retired” - “Self-employed” | -0.16 | 0.4366 | 0.4366 | 1.0000 |
|  |  | “Full-time” - “Unemployed/job-seeking” | 1.38 | 0.0844 | 0.0844 | 1.0000 |
|  |  | “Homemaker” - “Unemployed/job-seeking” | 2.02 | 0.0219 | 0.0219 | 0.4609 |
|  |  | “In education” - “Unemployed/job-seeking” | 0.41 | 0.3396 | 0.3396 | 1.0000 |
|  |  | “Part-time” - “Unemployed/job-seeking” | 0.85 | 0.1985 | 0.1985 | 1.0000 |
|  |  | “Retired” - “Unemployed/job-seeking” | -0.61 | 0.2710 | 0.2710 | 1.0000 |
|  |  | “Self-employed” - “Unemployed/job-seeking” | -0.57 | 0.2848 | 0.2848 | 1.0000 |
|  | **Smartphone use type** | |  |  |  |  |
|  |  | “Both equally” - “Mainly private” | -0.71 | 0.2388 | 0.2388 | 1.0000 |
|  |  | “Both equally” - “Mainly work” | 2.74 | 0.0031 | 0.0031 | 0.0311 |
|  |  | “Mainly private” - “Mainly work” | 3.08 | 0.0010 | 0.0010 | 0.0104 |
|  |  | “Both equally” - “Private only” | -1.02 | 0.1535 | 0.1535 | 1.0000 |
|  |  | “Mainly private” - “Private only” | -0.41 | 0.3416 | 0.3416 | 1.0000 |
|  |  | “Mainly work” - “Private only” | -3.19 | 0.0007 | 0.0007 | 0.0070 |
|  |  | “Both equally” - “Work only” | 1.57 | 0.0582 | 0.0582 | 0.5816 |
|  |  | “Mainly private” - “Work only” | 1.78 | 0.0376 | 0.0376 | 0.3760 |
|  |  | “Mainly work” - “Work only” | -0.34 | 0.3658 | 0.3658 | 1.0000 |
|  |  | “Private only” - “Work only” | 1.86 | 0.0318 | 0.0318 | 0.3180 |
| **Type Unknown** | | |  |  |  |  |
|  | **Gender** | |  |  |  |  |
|  |  | "Female" - "Male" | 2.39 | 0.0085 | 0.0085 | 0.0085 |
|  | **Smartphone use type** | |  |  |  |  |
|  |  | “Both equally” - “Mainly private” | -1.05 | 0.1478 | 0.1478 | 1.0000 |
|  |  | “Both equally” - “Mainly work” | 1.49 | 0.0681 | 0.0681 | 0.6809 |
|  |  | “Mainly private” - “Mainly work” | 1.92 | 0.0277 | 0.0277 | 0.2769 |
|  |  | “Both equally” - “Private only” | 0.31 | 0.3798 | 0.3798 | 1.0000 |
|  |  | “Mainly private” - “Private only” | 1.98 | 0.0238 | 0.0238 | 0.2377 |
|  |  | “Mainly work” - “Private only” | -1.44 | 0.0756 | 0.0756 | 0.7562 |
|  |  | “Both equally” - “Work only” | 1.91 | 0.0282 | 0.0282 | 0.2816 |
|  |  | “Mainly private” - “Work only” | 2.21 | 0.0136 | 0.0136 | 0.1356 |
|  |  | “Mainly work” - “Work only” | 0.69 | 0.2437 | 0.2437 | 1.0000 |
|  |  | “Private only” - “Work only” | 1.87 | 0.0310 | 0.0310 | 0.3099 |
