# Supplementary material for: Smartphone Usage Patterns and Sleep Behavior in Demographic Groups: Retrospective Observational Study
Source: J Med Internet Res. 2025 Jul 3;27:e60423. doi: 10.2196/60423 (PMC12271961; doi:10.2196/60423)
Supplement: Multimedia Appendix 5 [file jmir_v27i1e60423_app5.docx]

Multimedia Appendix 5. Dunn's Test of Differences in Proportion of Daily Usage of Smartphone Application Across Various Groups

| Indicator | Group Category | Comparison | Z Value | *P* Value Uncorrected | *P* Value Before | *P* Value Adjusted |
| --- | --- | --- | --- | --- | --- | --- |
| **Type A** | | |  |  |  |  |
|  | **Gender** | |  |  |  |  |
|  |  | “Female” - “Male” | -3.46 | 0.0003 | 0.0003 | 0.0003 |
|  | **Age** | |  |  |  |  |
|  |  | “Less than 18 years” - “60 years or older “ | -0.52 | 0.3007 | 0.3007 | 1.0000 |
|  |  | “Less than 18 years” - “18 years or older < 35 years” | 0.40 | 0.3455 | 0.3455 | 1.0000 |
|  |  | “60 years or older” - “18 years or older < 35 years” | 1.22 | 0.1106 | 0.1106 | 0.6636 |
|  |  | “Less than 18 years” - “35 years or older < 60 years” | -0.44 | 0.3314 | 0.3314 | 1.0000 |
|  |  | “60 years or older” - “35 years or older < 60 years” | 0.28 | 0.3914 | 0.3914 | 1.0000 |
| 、 |  | “18 years or older < 35 years” - “35 years or older < 60 years” | -3.49 | 0.0002 | 0.0002 | 0.0014 |
|  | **Highest degree** | |  |  |  |  |
|  |  | “Bachelor’s degree” - “Doctorate” | 0.24 | 0.4057 | 0.4057 | 1.0000 |
|  |  | “Bachelor’s degree” - “High school degree or equivalent” | 3.99 | 0.0000 | 0.0000 | 0.0005 |
|  |  | “Doctorate” - “High school degree or equivalent” | 0.84 | 0.1993 | 0.1993 | 1.0000 |
|  |  | “Bachelor’s degree” - “Master’s degree” | -1.60 | 0.0545 | 0.0545 | 0.8178 |
|  |  | “Doctorate” - “Master’s degree” | -0.83 | 0.2022 | 0.2022 | 1.0000 |
|  |  | “High school degree or equivalent” - “Master’s degree” | -5.15 | 0.0000 | 0.0000 | 0.0000 |
|  |  | “Bachelor’s degree” - “No formal qualification” | 2.29 | 0.0111 | 0.0111 | 0.1659 |
|  |  | “Doctorate” - “No formal qualification” | 1.53 | 0.0630 | 0.0630 | 0.9452 |
|  |  | “High school degree or equivalent” - “No formal qualification” | 1.29 | 0.0982 | 0.0982 | 1.0000 |
|  |  | “Master’s degree” - “No formal qualification” | 2.83 | 0.0024 | 0.0024 | 0.0353 |
|  |  | “Bachelor’s degree” - “Secondary education” | 1.35 | 0.0880 | 0.0880 | 1.0000 |
|  |  | “Doctorate” - “Secondary education” | 0.30 | 0.3820 | 0.3820 | 1.0000 |
|  |  | “High school degree or equivalent” - “Secondary education” | -1.47 | 0.0705 | 0.0705 | 1.0000 |
|  |  | “Master’s degree” - “Secondary education” | 2.60 | 0.0047 | 0.0047 | 0.0701 |
|  |  | “No formal qualification” - “Secondary education” | -1.72 | 0.0423 | 0.0423 | 0.6352 |
|  | **Employment status** | |  |  |  |  |
|  |  | “Full-time” - “Homemaker” | 0.33 | 0.3706 | 0.3706 | 1.0000 |
|  |  | “Full-time” - “In education” | 1.42 | 0.0777 | 0.0777 | 1.0000 |
|  |  | “Homemaker” - “In education” | 0.06 | 0.4766 | 0.4766 | 1.0000 |
|  |  | “Full-time” - “Part-time” | -1.97 | 0.0244 | 0.0244 | 0.5117 |
|  |  | “Homemaker” - “Part-time” | -1.05 | 0.1480 | 0.1480 | 1.0000 |
|  |  | “In education” - “Part-time” | -3.33 | 0.0004 | 0.0004 | 0.0093 |
|  |  | “Full-time” - “Retired” | -2.10 | 0.0180 | 0.0180 | 0.3790 |
|  |  | “Homemaker” - “Retired” | -1.70 | 0.0442 | 0.0442 | 0.9288 |
|  |  | “In education” - “Retired” | -2.56 | 0.0052 | 0.0052 | 0.1098 |
|  |  | “Part-time” - “Retired” | -1.260 | 0.1037 | 0.1037 | 1.0000 |
|  |  | “Full-time” - “Self-employed” | -1.01 | 0.1552 | 0.1552 | 1.0000 |
|  |  | “Homemaker” - “Self-employed” | -0.84 | 0.1998 | 0.1998 | 1.0000 |
|  |  | “In education” - “Self-employed” | -1.71 | 0.0439 | 0.0439 | 0.9221 |
|  |  | “Part-time” - “Self-employed” | 0.18 | 0.4293 | 0.4293 | 1.0000 |
|  |  | “Retired” - “Self-employed” | 1.23 | 0.1098 | 0.1098 | 1.0000 |
|  |  | “Full-time” - “Unemployed/job-seeking” | 0.65 | 0.2570 | 0.2570 | 1.0000 |
|  |  | “Homemaker” - “Unemployed/job-seeking” | 0.17 | 0.4308 | 0.4308 | 1.0000 |
|  |  | “In education” - “Unemployed/job-seeking” | 0.20 | 0.4220 | 0.4220 | 1.0000 |
|  |  | “Part-time” - “Unemployed/job-seeking” | 1.48 | 0.0692 | 0.0692 | 1.0000 |
|  |  | “Retired”- “Unemployed/job-seeking” | 2.05 | 0.0203 | 0.0203 | 0.4260 |
|  |  | “Self-employed” - “Unemployed/job-seeking” | 1.18 | 0.1193 | 0.1193 | 1.0000 |
|  | **Smartphone use type** | |  |  |  |  |
|  |  | “Both equally” - “Mainly private” | 1.12 | 0.1312 | 0.1312 | 1.0000 |
|  |  | “Both equally” - “Mainly work” | -1.38 | 0.0845 | 0.0845 | 0.8449 |
|  |  | “Mainly private” - “Mainly work” | -1.82 | 0.0340 | 0.0340 | 0.3401 |
|  |  | “Both equally” - “Private only” | 2.07 | 0.0195 | 0.0195 | 0.1945 |
|  |  | “Mainly private” - “Private only” | 1.29 | 0.0984 | 0.0984 | 0.9840 |
|  |  | “Mainly work” - “Private only” | 2.15 | 0.0157 | 0.0157 | 0.1571 |
|  |  | “Both equally” - “Work only” | -2.02 | 0.0218 | 0.0218 | 0.2185 |
|  |  | “Mainly private” - “Work only” | -2.34 | 0.0097 | 0.0097 | 0.0966 |
|  |  | “Mainly work” - “Work only” | -0.85 | 0.1964 | 0.1964 | 1.0000 |
|  |  | “Private only” - “Work only” | -2.57 | 0.0051 | 0.0051 | 0.0507 |
| **Type B** | | |  |  |  |  |
|  | **Age** | |  |  |  |  |
|  |  | “Less than 18 years” - “60 years or older “ | -0.39 | 0.3482 | 0.3482 | 1.0000 |
|  |  | “Less than 18 years” - “18 years or older < 35 years” | -0.36 | 0.3587 | 0.3587 | 1.0000 |
|  |  | “60 years or older” - “18 years or older < 35 years” | 0.18 | 0.4294 | 0.4294 | 1.0000 |
|  |  | “Less than 18 years” - “35 years or older < 60 years” | 0.60 | 0.2735 | 0.2735 | 1.0000 |
|  |  | “60 years or older” - “35 years or older < 60 years” | 1.24 | 0.1080 | 0.1080 | 0.6479 |
|  |  | “18 years or older < 35 years” - “35 years or older < 60 years” | 4.05 | 0.0000 | 0.0000 | 0.0002 |
|  | **Highest degree** | |  |  |  |  |
|  |  | “Bachelor’s degree” - “Doctorate” | -0.17 | 0.4331 | 0.4331 | 1.0000 |
|  |  | “Bachelor’s degree” - “High school degree or equivalent” | -1.74 | 0.0409 | 0.0409 | 0.6128 |
|  |  | “Doctorate” - “High school degree or equivalent” | -0.30 | 0.3812 | 0.3812 | 1.0000 |
|  |  | “Bachelor’s degree” - “Master’s degree” | 2.23 | 0.0128 | 0.0128 | 0.1926 |
|  |  | “Doctorate” - “Master’s degree” | 1.00 | 0.1587 | 0.1587 | 1.0000 |
|  |  | “High school degree or equivalent” - “Master’s degree” | 4.04 | 0.0000 | 0.0000 | 0.0004 |
|  |  | “Bachelor’s degree” - “No formal qualification” | 0.62 | 0.2669 | 0.2669 | 1.0000 |
|  |  | “Doctorate” - “No formal qualification” | 0.58 | 0.2804 | 0.2804 | 1.0000 |
|  |  | “High school degree or equivalent” - “No formal qualification” | 1.09 | 0.1387 | 0.1387 | 1.0000 |
|  |  | “Master’s degree” - “No formal qualification” | -0.19 | 0.4263 | 0.4263 | 1.0000 |
|  |  | “Bachelor’s degree” - “Secondary education” | 1.44 | 0.0750 | 0.0750 | 1.0000 |
|  |  | “Doctorate” - “Secondary education” | 0.73 | 0.2325 | 0.2325 | 1.0000 |
|  |  | “High school degree or equivalent” - “Secondary education” | 2.96 | 0.0015 | 0.0015 | 0.0227 |
|  |  | “Master’s degree” - “Secondary education” | -0.60 | 0.2743 | 0.2743 | 1.0000 |
|  |  | “No formal qualification” - “Secondary education” | -0.06 | 0.4741 | 0.4741 | 1.0000 |
|  | **Employment status** | |  |  |  |  |
|  |  | “Full-time” - “Homemaker” | 0.22 | 0.4129 | 0.4129 | 1.0000 |
|  |  | “Full-time” - “In education” | -2.53 | 0.0056 | 0.0056 | 0.1185 |
|  |  | “Homemaker” - “In education” | -0.92 | 0.1777 | 0.1777 | 1.0000 |
|  |  | “Full-time” - “Part-time” | 1.41 | 0.0787 | 0.0787 | 1.0000 |
|  |  | “Homemaker” - “Part-time” | 0.30 | 0.3816 | 0.3816 | 1.0000 |
|  |  | “In education” - “Part-time” | 3.61 | 0.0002 | 0.0002 | 0.0032 |
|  |  | “Full-time” - “Retired” | 0.28 | 0.3882 | 0.3882 | 1.0000 |
|  |  | “Homemaker” - “Retired” | 0.03 | 0.4882 | 0.4882 | 1.0000 |
|  |  | “In education” - “Retired” | 1.06 | 0.1448 | 0.1448 | 1.0000 |
|  |  | “Part-time” - “Retired” | -0.29 | 0.3869 | 0.3869 | 1.0000 |
|  |  | “Full-time” - “Self-employed” | 2.64 | 0.0042 | 0.0042 | 0.0881 |
|  |  | “Homemaker” - “Self-employed” | 1.23 | 0.1087 | 0.1087 | 1.0000 |
|  |  | “In education” - “Self-employed” | 3.90 | 0.0000 | 0.0000 | 0.0010 |
|  |  | “Part-time” - “Self-employed” | 1.69 | 0.0458 | 0.0458 | 0.9612 |
|  |  | “Retired” - “Self-employed” | 1.29 | 0.0991 | 0.0991 | 1.0000 |
|  |  | “Full-time” - “Unemployed/job-seeking” | -2.74 | 0.0030 | 0.0030 | 0.0639 |
|  |  | “Homemaker” - “Unemployed/job-seeking” | -2.00 | 0.0228 | 0.0228 | 0.4798 |
|  |  | “In education” - “Unemployed/job-seeking” | -1.96 | 0.0251 | 0.0251 | 0.5266 |
|  |  | “Part-time” - “Unemployed/job-seeking” | -3.28 | 0.0005 | 0.0005 | 0.0110 |
|  |  | “Retired” - “Unemployed/job-seeking” | -2.14 | 0.0161 | 0.0161 | 0.3371 |
|  |  | “Self-employed” - “Unemployed/job-seeking” | -3.95 | 0.0000 | 0.0000 | 0.0008 |
|  | **Smartphone use type** | |  |  |  |  |
|  |  | “Both equally” - “Mainly private” | -2.64 | 0.0042 | 0.0042 | 0.0418 |
|  |  | “Both equally” - “Mainly work” | 2.83 | 0.0023 | 0.0023 | 0.0235 |
|  |  | “Mainly private” - “Mainly work” | 3.87 | 0.0001 | 0.0001 | 0.0005 |
|  |  | “Both equally” - “Private only” | -2.24 | 0.0125 | 0.0125 | 0.1250 |
|  |  | “Mainly private” - “Private only” | 0.70 | 0.2425 | 0.2425 | 1.0000 |
|  |  | “Mainly work” - “Private only” | -3.72 | 0.0001 | 0.0001 | 0.0010 |
|  |  | “Both equally” - “Work only” | 1.64 | 0.0503 | 0.0503 | 0.5029 |
|  |  | “Mainly private” - “Work only” | 2.35 | 0.0094 | 0.0094 | 0.0936 |
|  |  | “Mainly work” - “Work only” | -0.34 | 0.3676 | 0.3676 | 1.0000 |
|  |  | “Private only” - “Work only” | 2.23 | 0.0127 | 0.0127 | 0.1275 |
| **Type D** | | |  |  |  |  |
|  | **Gender** | |  |  |  |  |
|  |  | “Female” - “Male” | 4.4921 | 0.0000 | 0.0000 | 0.0000 |
|  | **Smartphone use type** | |  |  |  |  |
|  |  | “Both equally” - “Mainly private” | -1.56 | 0.0596 | 0.0596 | 0.5957 |
|  |  | “Both equally” - “Mainly work” | 2.74 | 0.0030 | 0.0030 | 0.0304 |
|  |  | “Mainly private” - “Mainly work” | 3.39 | 0.0003 | 0.0003 | 0.0034 |
|  |  | “Both equally” - “Private only” | -1.32 | 0.0935 | 0.0935 | 0.9353 |
|  |  | “Mainly private” - “Private only” | 0.42 | 0.3373 | 0.3373 | 1.0000 |
|  |  | “Mainly work” - “Private only” | -3.31 | 0.0005 | 0.0005 | 0.0047 |
|  |  | “Both equally” - “Work only” | 2.70 | 0.0034 | 0.0034 | 0.0345 |
|  |  | “Mainly private” - “Work only” | 3.15 | 0.0008 | 0.0008 | 0.0082 |
|  |  | “Mainly work” - “Work only” | 0.60 | 0.2746 | 0.2746 | 1.0000 |
|  |  | “Private only” - “Work only” | 3.08 | 0.0010 | 0.0010 | 0.0103 |
| **Type E** | | |  |  |  |  |
|  | **Gender** | |  |  |  |  |
|  |  | “Female” - “Male” | 2.76 | 0.0029 | 0.0029 | 0.0029 |
|  | **Employment status** | |  |  |  |  |
|  |  | “Full-time” - “Homemaker” | -1.29 | 0.0992 | 0.0992 | 1.0000 |
|  |  | “Full-time” - “In education” | 4.74 | 0.0000 | 0.0000 | 0.0000 |
|  |  | “Homemaker” - “In education” | 2.61 | 0.0045 | 0.0045 | 0.0938 |
|  |  | “Full-time” - “Part-time” | 1.41 | 0.0786 | 0.0786 | 1.0000 |
|  |  | “Homemaker” - “Part-time” | 1.78 | 0.0376 | 0.0376 | 0.7902 |
|  |  | “In education” - “Part-time” | -2.27 | 0.0115 | 0.0115 | 0.2412 |
|  |  | “Full-time” - “Retired” | 1.92 | 0.0272 | 0.0272 | 0.5716 |
|  |  | “Homemaker” - “Retired” | 2.31 | 0.0104 | 0.0104 | 0.2193 |
|  |  | “In education” - “Retired” | 0.51 | 0.3048 | 0.3048 | 1.0000 |
|  |  | “Part-time” - “Retired” | 1.31 | 0.0944 | 0.0944 | 1.0000 |
|  |  | “Full-time” - “Self-employed” | 2.46 | 0.0070 | 0.0070 | 0.1467 |
|  |  | “Homemaker” - “Self-employed” | 2.47 | 0.0067 | 0.0067 | 0.1414 |
|  |  | “In education” - “Self-employed” | 0.35 | 0.3624 | 0.3624 | 1.0000 |
|  |  | “Part-time” - “Self-employed” | 1.52 | 0.0645 | 0.0645 | 1.0000 |
|  |  | “Retired” - “Self-employed” | -0.24 | 0.4058 | 0.4058 | 1.0000 |
|  |  | “Full-time” - “Unemployed/job-seeking” | 2.74 | 0.0031 | 0.0031 | 0.0645 |
|  |  | “Homemaker” - “Unemployed/job-seeking” | 2.83 | 0.0023 | 0.0023 | 0.0482 |
|  |  | “In education” - “Unemployed/job-seeking” | 1.23 | 0.1095 | 0.1095 | 1.0000 |
|  |  | “Part-time” - “Unemployed/job-seeking” | 2.06 | 0.0197 | 0.0197 | 0.4139 |
|  |  | “Retired” - “Unemployed/job-seeking” | 0.47 | 0.3202 | 0.3202 | 1.0000 |
|  |  | “Self-employed” - “Unemployed/job-seeking” | 0.82 | 0.2069 | 0.2069 | 1.0000 |
|  | **Smartphone use type** | |  |  |  |  |
|  |  | “Both equally” - “Mainly private” | -0.41 | 0.3406 | 0.3406 | 1.0000 |
|  |  | “Both equally” - “Mainly work” | 1.93 | 0.0268 | 0.0268 | 0.2681 |
|  |  | “Mainly private” - “Mainly work” | 2.14 | 0.0162 | 0.0162 | 0.1623 |
|  |  | “Both equally” - “Private only” | -0.34 | 0.3666 | 0.3666 | 1.0000 |
|  |  | “Mainly private” - “Private only” | 0.12 | 0.4520 | 0.4520 | 1.0000 |
|  |  | “Mainly work” - “Private only” | -2.12 | 0.0171 | 0.0171 | 0.1707 |
|  |  | “Both equally” - “Work only” | 2.79 | 0.0026 | 0.0026 | 0.0262 |
|  |  | “Mainly private” - “Work only” | 2.94 | 0.0016 | 0.0016 | 0.0163 |
|  |  | “Mainly work” - “Work only” | 1.17 | 0.1215 | 0.1215 | 1.0000 |
|  |  | “Private only” - “Work only” | 2.93 | 0.0017 | 0.0017 | 0.0171 |
| **Type Unknown** | | |  |  |  |  |
|  | **Age** | |  |  |  |  |
|  |  | “Less than 18 years” - “60 years or older “ | 1.64 | 0.0505 | 0.0505 | 0.3032 |
|  |  | “Less than 18 years” - “18 years or older < 35 years” | 2.57 | 0.0051 | 0.0051 | 0.0305 |
|  |  | “60 years or older” - “18 years or older < 35 years” | 0.42 | 0.3355 | 0.3355 | 1.0000 |
|  |  | “Less than 18 years” - “35 years or older < 60 years” | 1.65 | 0.0491 | 0.0491 | 0.2945 |
|  |  | “60 years or older” - “35 years or older < 60 years” | -0.55 | 0.2917 | 0.2917 | 1.0000 |
|  |  | “18 years or older < 35 years” - “35 years or older < 60 years” | -3.67 | 0.0001 | 0.0001 | 0.0007 |
|  | **Highest degree** | |  |  |  |  |
|  |  | “Bachelor’s degree” - “Doctorate” | -2.08 | 0.0186 | 0.0186 | 0.2794 |
|  |  | “Bachelor’s degree” - “High school degree or equivalent” | 0.88 | 0.1900 | 0.1900 | 1.0000 |
|  |  | “Doctorate” - “High school degree or equivalent” | 2.36 | 0.0090 | 0.0090 | 0.1357 |
|  |  | “Bachelor’s degree” - “Master’s degree” | -0.74 | 0.2289 | 0.2289 | 1.0000 |
|  |  | “Doctorate” - “Master’s degree” | 1.77 | 0.0383 | 0.0383 | 0.5752 |
|  |  | “High school degree or equivalent” - “Master’s degree” | -1.59 | 0.0561 | 0.0561 | 0.8422 |
|  |  | “Bachelor’s degree” - “No formal qualification” | -0.04 | 0.4829 | 0.4829 | 1.0000 |
|  |  | “Doctorate” - “No formal qualification” | 1.45 | 0.0740 | 0.0740 | 1.0000 |
|  |  | “High school degree or equivalent” - “No formal qualification” | -0.27 | 0.3928 | 0.3928 | 1.0000 |
|  |  | “Master’s degree” - “No formal qualification” | 0.22 | 0.4116 | 0.4116 | 1.0000 |
|  |  | “Bachelor’s degree” - “Secondary education” | -1.37 | 0.0852 | 0.0852 | 1.0000 |
|  |  | “Doctorate” - “Secondary education” | 1.49 | 0.0681 | 0.0681 | 1.0000 |
|  |  | “High school degree or equivalent” - “Secondary education” | -2.23 | 0.0127 | 0.0127 | 0.1912 |
|  |  | “Master’s degree” - “Secondary education” | -0.61 | 0.2707 | 0.2707 | 1.0000 |
|  |  | “No formal qualification” - “Secondary education” | -0.48 | 0.3173 | 0.3173 | 1.0000 |
|  | **Employment status** | |  |  |  |  |
|  |  | “Full-time” - “Homemaker” | -0.22 | 0.4113 | 0.4113 | 1.0000 |
|  |  | “Full-time” - “In education” | 2.58 | 0.0050 | 0.0050 | 0.1050 |
|  |  | “Homemaker” - “In education” | 0.94 | 0.1736 | 0.1736 | 1.0000 |
|  |  | “Full-time” - “Part-time” | -0.93 | 0.1770 | 0.1770 | 1.0000 |
|  |  | “Homemaker” - “Part-time” | -0.12 | 0.4526 | 0.4526 | 1.0000 |
|  |  | “In education” - “Part-time” | -3.11 | 0.0009 | 0.0009 | 0.0199 |
|  |  | “Full-time” - “Retired” | -1.28 | 0.0994 | 0.0994 | 1.0000 |
|  |  | “Homemaker” - “Retired” | -0.72 | 0.2357 | 0.2357 | 1.0000 |
|  |  | “In education” - “Retired” | -2.09 | 0.0184 | 0.0184 | 0.3865 |
|  |  | “Part-time” - “Retired” | -0.89 | 0.1880 | 0.1880 | 1.0000 |
|  |  | “Full-time” - “Self-employed” | -1.12 | 0.1310 | 0.1310 | 1.0000 |
|  |  | “Homemaker” - “Self-employed” | -0.41 | 0.3412 | 0.3412 | 1.0000 |
|  |  | “In education” - “Self-employed” | -2.35 | 0.0093 | 0.0093 | 0.1960 |
|  |  | “Part-time” - “Self-employed” | -0.53 | 0.2982 | 0.2982 | 1.0000 |
|  |  | “Retired” - “Self-employed” | 0.46 | 0.3221 | 0.3221 | 1.0000 |
|  |  | “Full-time” - “Unemployed/job-seeking” | 2.00 | 0.0226 | 0.0226 | 0.4745 |
|  |  | “Homemaker” - “Unemployed/job-seeking” | 1.51 | 0.0657 | 0.0657 | 1.0000 |
|  |  | “In education” - “Unemployed/job-seeking” | 1.19 | 0.1168 | 0.1168 | 1.0000 |
|  |  | “Part-time” - “Unemployed/job-seeking” | 2.35 | 0.0094 | 0.0094 | 0.1983 |
|  |  | “Retired” - “Unemployed/job-seeking” | 2.38 | 0.0086 | 0.0086 | 0.1809 |
|  |  | “Self-employed” - “Unemployed/job-seeking” | 2.39 | 0.0084 | 0.0084 | 0.1758 |
|  | **Smartphone use type** | |  |  |  |  |
|  |  | “Both equally” - “Mainly private” | 1.82 | 0.0345 | 0.0345 | 0.3453 |
|  |  | “Both equally” - “Mainly work” | -1.80 | 0.0363 | 0.0363 | 0.3633 |
|  |  | “Mainly private” - “Mainly work” | -2.51 | 0.0060 | 0.0060 | 0.0603 |
|  |  | “Both equally” - “Private only” | 2.22 | 0.0132 | 0.0132 | 0.1316 |
|  |  | “Mainly private” - “Private only” | 0.48 | 0.3143 | 0.3143 | 1.0000 |
|  |  | “Mainly work” - “Private only” | 2.64 | 0.0041 | 0.0041 | 0.0413 |
|  |  | “Both equally” - “Work only” | -1.94 | 0.0259 | 0.0259 | 0.2589 |
|  |  | “Mainly private” - “Work only” | -2.45 | 0.0072 | 0.0072 | 0.0721 |
|  |  | “Mainly work” - “Work only” | -0.54 | 0.2945 | 0.2945 | 1.0000 |
|  |  | “Private only” - “Work only” | -2.54 | 0.0056 | 0.0056 | 0.0559 |
