# Supplementary material for: Smartphone Usage Patterns and Sleep Behavior in Demographic Groups: Retrospective Observational Study
Source: J Med Internet Res. 2025 Jul 3;27:e60423. doi: 10.2196/60423 (PMC12271961; doi:10.2196/60423)
Supplement: Multimedia Appendix 6 [file jmir_v27i1e60423_app6.docx]

Multimedia Appendix 6. Dunn's Test of Differences in Daily Duration of Smartphone Usage Across Various Groups

| Group Category | Comparison | Z Value | *P* Value Uncorrected | *P* Value Before | *P* Value Adjusted |
| --- | --- | --- | --- | --- | --- |
| **Gender** | |  |  |  |  |
|  | "Female" - "Male" | 3.93 | 0.0000 | 0.0000 | 0.0000 |
| **Age** | |  |  |  |  |
|  | " Less than 18 years " - "60 years or older " | 1.34 | 0.0905 | 0.0905 | 0.5428 |
|  | " Less than 18 years " - "18 years or older < 35 years " | -0.88 | 0.1886 | 0.1886 | 1.0000 |
|  | "60 years or older " - "18 years or older < 35 years " | -2.98 | 0.0014 | 0.0014 | 0.0086 |
|  | " Less than 18 years " - "35 years or older < 60years " | -0.30 | 0.3828 | 0.3828 | 1.0000 |
|  | "60 years or older " - "35 years or older < 60years " | -2.28 | 0.0113 | 0.0113 | 0.0681 |
|  | "18 years or older < 35 years " - "35 years or older < 60years " | 2.40 | 0.0082 | 0.0082 | 0.049 |
| **Highest degree** | |  |  |  |  |
|  | "Bachelor’s degree " - "Doctorate" | 0.86 | 0.1946 | 0.1946 | 1.0000 |
|  | "Bachelor’s degree " - "High school degree or equivalent" | -1.99 | 0.0231 | 0.0231 | 0.346 |
|  | "Doctorate" - "High school degree or equivalent" | -1.42 | 0.0776 | 0.0776 | 1.0000 |
|  | "Bachelor’s degree " - "Master’s degree" | 2.51 | 0.0060 | 0.006 | 0.0894 |
|  | "Doctorate" - "Master’s degree" | 0.09 | 0.4628 | 0.4628 | 1.0000 |
|  | "High school degree or equivalent" - "Master’s degree" | 4.57 | 0.0000 | 0.0000 | 0.0000 |
|  | "Bachelor’s degree " - "No formal qualification" | -0.63 | 0.2655 | 0.2655 | 1.0000 |
|  | "Doctorate" - "No formal qualification" | -1.08 | 0.1408 | 0.1408 | 1.0000 |
|  | "High school degree or equivalent" - "No formal qualification" | -0.12 | 0.4524 | 0.4524 | 1.0000 |
|  | "Master’s degree" - "No formal qualification" | -1.52 | 0.0647 | 0.0647 | 0.9698 |
|  | "Bachelor’s degree " - "Secondary education" | 0.78 | 0.2180 | 0.2180 | 1.0000 |
|  | "Doctorate" - "Secondary education" | -0.53 | 0.2972 | 0.2972 | 1.0000 |
|  | "High school degree or equivalent" - "Secondary education" | 2.40 | 0.0082 | 0.0082 | 0.1228 |
|  | "Master’s degree" - "Secondary education" | -1.44 | 0.0746 | 0.0746 | 1.0000 |
|  | "No formal qualification" - "Secondary education" | 0.91 | 0.1826 | 0.1826 | 1.0000 |
| **Employment status** | |  |  |  |  |
|  | "Full-time" - "Homemaker" | -1.83 | 0.0334 | 0.0334 | 0.7005 |
|  | "Full-time" - "In education" | -2.64 | 0.0042 | 0.0042 | 0.0879 |
|  | "Homemaker" - "In education" | 1.13 | 0.1298 | 0.1298 | 1.0000 |
|  | "Full-time" - "Part-time" | 1.76 | 0.0393 | 0.0393 | 0.8249 |
|  | "Homemaker" - "Part-time" | 2.44 | 0.0073 | 0.0073 | 0.1534 |
|  | "In education" - "Part-time" | 4.07 | 0.0000 | 0.0000 | 5e-04 |
|  | "Full-time" - "Retired" | 3.64 | 0.0001 | 0.0001 | 0.0029 |
|  | "Homemaker" - "Retired" | 3.92 | 0.0000 | 0.0000 | 9e-04 |
|  | "In education" - "Retired" | 4.50 | 0.0000 | 0.0000 | 1e-04 |
|  | "Part-time" - "Retired" | 2.85 | 0.0022 | 0.0022 | 0.0454 |
|  | "Full-time" - "Self-employed" | 1.83 | 0.0338 | 0.0338 | 0.7105 |
|  | "Homemaker" - "Self-employed" | 2.61 | 0.0045 | 0.0045 | 0.0939 |
|  | "In education" - "Self-employed" | 3.11 | 0.0009 | 0.0009 | 0.0196 |
|  | "Part-time" - "Self-employed" | 0.72 | 0.2364 | 0.2364 | 1.0000 |
|  | "Retired" - "Self-employed" | -2.09 | 0.0181 | 0.0181 | 0.3810 |
|  | "Full-time" - "Unemployed/job-seeking" | -2.30 | 0.0108 | 0.0108 | 0.2263 |
|  | "Homemaker" - "Unemployed/job-seeking" | -0.09 | 0.4656 | 0.4656 | 1.0000 |
|  | "In education" - "Unemployed/job-seeking" | -1.47 | 0.0705 | 0.0705 | 1.0000 |
|  | "Part-time" - "Unemployed/job-seeking" | -2.99 | 0.0014 | 0.0014 | 0.0290 |
|  | "Retired" - ""Unemployed/job-seeking" | -4.38 | 0.0000 | 0.0000 | 1e-04 |
|  | "Self-employed" - "Unemployed/job-seeking"" | -3.08 | 0.0010 | 0.0010 | 0.0219 |
| **Smartphone use type** | |  |  |  |  |
|  | "Both equally" - "Mainly private" | -1.89 | 0.0291 | 0.0291 | 0.2911 |
|  | "Both equally" - "Mainly work" | 4.14 | 0.0000 | 0.0000 | 2e-04 |
|  | "Mainly private" - "Mainly work" | 4.96 | 0.0000 | 0.0000 | 0.0000 |
|  | "Both equally" - "Private only" | -1.05 | 0.1479 | 0.1479 | 1.0000 |
|  | "Mainly private" - "Private only" | 1.31 | 0.0958 | 0.0958 | 0.9575 |
|  | "Mainly work" - "Private only" | -4.65 | 0.0000 | 0.0000 | 0.0000 |
|  | "Both equally" - "Work only" | 3.33 | 0.0004 | 0.0004 | 0.0044 |
|  | "Mainly private" - "Work only" | 3.87 | 0.0001 | 0.0001 | 5e-04 |
|  | "Mainly work" - "Work only" | 0.28 | 0.3910 | 0.3910 | 11.0000 |
|  | "Private only" - "Work only" | 3.65 | 0.0001 | 0.0001 | 0.0013 |
