# Supplementary material for: Smartphone Usage Patterns and Sleep Behavior in Demographic Groups: Retrospective Observational Study
Source: J Med Internet Res. 2025 Jul 3;27:e60423. doi: 10.2196/60423 (PMC12271961; doi:10.2196/60423)
Supplement: Multimedia Appendix 7 [file jmir_v27i1e60423_app7.docx]

Multimedia Appendix 7. Statistical Analysis of Nocturnal Smartphone Inactivity Data Across Various Groups

| Research object | | Participants with Nocturnal Smartphone Inactivity Below 6 Hours | | Participants with Nocturnal Smartphone Inactivity Exceeding 6 Hours | | | | |
| --- | --- | --- | --- | --- | --- | --- | --- | --- |
| Group Category | | Number of Participants, n (%) | *P*-value | Number of Participants | Daily Nocturnal Inactivity Duration on Days Exceeding 6 Hours of Phone Non-Use | | Proportion of Days with Exceeding 6 Hours of Nocturnal Smartphone Inactivity | |
|  |  |  |  |  | Median [Q1, Q3] (h) | *P*-value | Median [Q1, Q3] (%) | *P*-value |
| **Gender** | |  |  |  |  |  |  |  |
|  | Male(n=350) | 41 (12%) | .003 | 309 | 8.34 [7.84, 9.12] | .08 | 64.81[43.75, 80.92] | < .001 |
|  | Female(n=724) | 45 (6%) |  | 679 | 8.28 [7.77, 8.85] |  | 73.68 [50.44, 86.49] |  |
| **Age** | |  |  |  |  |  |  |  |
|  | Less than 18 years(n=8) | 1 (13%) | .54 | 7 | 8.92 [8.18, 11.27] | < .001 | 46.20 [24.04, 60.71] | < .001 |
|  | 18 years or older < 35 years(n=895) | 75 (8%) |  | 820 | 8.28 [7.77, 8.88] |  | 69.82 [47.12, 83.79] |  |
|  | 35 years or older < 60 years(n=161) | 10 (6%) |  | 151 | 8.30 [7.79, 9.23] |  | 80.93 [55.56, 91.93] |  |
|  | 60 years or older(n=10) | 0 (0%) |  | 10 | 10.81[9.83, 11.91] |  | 94.58 [81.88, 97.42] |  |
| **Highest degree** | |  |  |  |  |  |  |  |
|  | Doctorate(n=11) | 1 (9.%) | .97 | 10 | 8.58 [7.66, 9.39] | .24 | 81.27 [64.39, 91.55] | .001 |
|  | Master’s degree(n=116) | 10 (9%) |  | 106 | 8.28 [7.73, 9.29] |  | 79.13 [55.73, 90.91] |  |
|  | Bachelor’s degree(n=189) | 16 (8%) |  | 173 | 8.18 [7.73, 8.81] |  | 69.32 [47.83, 82.61] |  |
|  | Secondary education(n=96) | 6 (6%) |  | 90 | 8.42 [7.77, 9.47] |  | 75.28 [53.88, 90.52] |  |
|  | High school degree or equivalent (n=637) | 53 (8%) |  | 584 | 8.29 [7.83, 8.87] |  | 69.92 [46.40, 84.00] |  |
|  | No formal qualification(n=10) | 0 (0%) |  | 10 | 8.56 [7.60, 11.16] |  | 50.83 [26.20, 70.29] |  |
| **Employment status** | |  |  |  |  |  |  |  |
|  | In education(n=535) | 50 (9%) | .03 | 485 | 8.31 [7.82, 8.89] | < .001 | 68.97 [44.83, 83.33] | .002 |
|  | Unemployed job-seeking(n=20) | 0 (0.00%) |  | 20 | 8.97 [8.39, 11.16] |  | 33.33 [28.10, 83.81] |  |
|  | Part-time (n=149) | 12 (8.05%) |  | 137 | 8.22 [7.77, 8.80] |  | 76.30 [52.62, 88.40] |  |
|  | Full-time(n=267) | 11 (4.12%) |  | 256 | 8.15 [7.67, 8.82] |  | 74.45 [52.82, 86.78] |  |
|  | Self-employed(n=41) | 7 (17%) |  | 34 | 8.39 [7.87, 9.29] |  | 72.73 [51.39, 91.00] |  |
|  | Homemaker(n=14) | 1 (7%) |  | 13 | 8.70 [8.11, 9.61] |  | 69.05 [39.13, 86.36] |  |
|  | Retired(n=17) | 1 (6%) |  | 16 | 9.91 [8.50, 12.20] |  | 86.02 [69.06, 91.04] |  |
| **Smartphone use type** | |  |  |  |  |  |  |  |
|  | Both equally(n=139) | 15 (11%) | .15 | 124 | 8.28 [7.74, 8.99] | <0.001 | 69.53 [39.32, 82.69] | .17 |
|  | Mainly private(n=390) | 26 (7%) |  | 364 | 8.28 [7.78, 8.89] |  | 71.21 [49.61, 84.86] |  |
|  | Mainly work(n=14) | 1 (7%) |  | 13 | 14.42 [9.48, 15.94] |  | 57.14 [47.37, 65.48] |  |
|  | Private only(n=524) | 43 (8%) |  | 481 | 8.30 [7.77, 8.90] |  | 71.64 [50.37, 86.36] |  |
|  | Work only(n=7) | 2 (29%) |  | 5 | 13.94 [11.84, 14.54] |  | 74.71 [61.90, 75.61] |  |

Note: The values for ’Daily Nocturnal Inactivity Duration on Days Exceeding 6 Hours of Phone Non-Use’ and ’Proportion of Days with Exceeding 6 Hours of Nocturnal Smartphone Inactivity’ represent participant-level metrics summarized as median values with the first (Q1) and third (Q3) quartiles within each group. Each participant contributed one value per metric.
