# Supplementary material for: Smartphone Usage Patterns and Sleep Behavior in Demographic Groups: Retrospective Observational Study
Source: J Med Internet Res. 2025 Jul 3;27:e60423. doi: 10.2196/60423 (PMC12271961; doi:10.2196/60423)
Supplement: Multimedia Appendix 8 [file jmir_v27i1e60423_app8.docx]

Multimedia Appendix 8. Dunn's Test of Differences in Nocturnal Smartphone Inactivity Duration on Days Exceeding 6 Hours

| Group Category | Comparison | Z Value | *P* Value Uncorrected | *P* Value Before | *P* Value Adjusted |
| --- | --- | --- | --- | --- | --- |
| **Age** | |  |  |  |  |
|  | “Less than 18 years” - “60 years or older” | -1.34 | 0.0898 | 0.0898 | 0.5387 |
|  | “Less than 18 years” - “18 years or older < 35 years” | 1.46 | 0.0716 | 0.0716 | 0.4296 |
|  | “60 years or older” - “18 years or older < 35 years” | 3.83 | 0.0001 | 0.0001 | 0.0004 |
|  | “Less than 18 years” - “35 years or older < 60 years” | 1.14 | 0.1263 | 0.1263 | 0.7579 |
|  | “60 years or older” - “35 years or older < 60 years” | 3.38 | 0.0004 | 0.0004 | 0.0022 |
|  | “18 years or older < 35 years” - “35 years or older < 60 years” | -1.28 | 0.1009 | 0.1009 | 0.6056 |
| **Employment status** | |  |  |  |  |
|  | “Full-time” - “Homemaker” | -1.66 | 0.0485 | 0.0485 | 1.0000 |
|  | “Full-time” - “In education” | -1.97 | 0.0245 | 0.0245 | 0.5142 |
|  | “Homemaker” - “In education” | 1.14 | 0.1275 | 0.1275 | 1.0000 |
|  | “Full-time” - “Part-time” | -0.76 | 0.2250 | 0.2250 | 1.0000 |
|  | “Homemaker” - “Part-time” | 1.35 | 0.0886 | 0.0886 | 1.0000 |
|  | “In education” - “Part-time” | 0.74 | 0.2292 | 0.2292 | 1.0000 |
|  | “Full-time” - “Retired” | -3.64 | 0.0001 | 0.0001 | 0.0028 |
|  | “Homemaker” - “Retired” | -1.25 | 0.1056 | 0.1056 | 1.0000 |
|  | “In education” - “Retired” | -3.10 | 0.0010 | 0.0010 | 0.0206 |
|  | “Part-time” - “Retired” | -3.25 | 0.0006 | 0.0006 | 0.0122 |
|  | “Full-time” - “Self-employed” | -1.99 | 0.0231 | 0.0231 | 0.4847 |
|  | “Homemaker” - “Self-employed” | 0.33 | 0.3703 | 0.3703 | 1.0000 |
|  | “In education” - “Self-employed” | -1.19 | 0.1162 | 0.1162 | 1.0000 |
|  | “Part-time” - “Self-employed” | -1.48 | 0.0694 | 0.0694 | 1.0000 |
|  | “Retired” - “Self-employed” | 1.90 | 0.0290 | 0.0290 | 0.6086 |
|  | “Full-time” - “Unemployed/job-seeking” | -3.14 | 0.0008 | 0.0008 | 0.0174 |
|  | “Homemaker” - “Unemployed/job-seeking” | -0.72 | 0.2343 | 0.2343 | 1.0000 |
|  | “In education” - “Unemployed/job-seeking” | -2.53 | 0.0056 | 0.0056 | 0.1186 |
|  | “Part-time” - “Unemployed/job-seeking” | -2.71 | 0.0033 | 0.0033 | 0.0697 |
|  | “Retired” - “Unemployed/job-seeking” | 0.62 | 0.2670 | 0.2670 | 1.0000 |
|  | “Self-employed” - “Unemployed/job-seeking” | -1.30 | 0.0969 | 0.0969 | 1.0000 |
| **Smartphone use type** | |  |  |  |  |
|  | “Both equally” - “Mainly private” | 0.52 | 0.3020 | 0.3020 | 1.0000 |
|  | “Both equally” - “Mainly work” | -3.67 | 0.0001 | 0.0001 | 0.0012 |
|  | “Mainly private” - “Mainly work” | -3.98 | 0.0000 | 0.0000 | 0.0003 |
|  | “Both equally” - “Private only” | 0.45 | 0.3249 | 0.3249 | 1.0000 |
|  | “Mainly private” - “Private only” | -0.12 | 0.4531 | 0.4531 | 1.0000 |
|  | “Mainly work” - “Private only” | 3.97 | 0.0000 | 0.0000 | 0.0004 |
|  | “Both equally” - “Work only” | -2.68 | 0.0037 | 0.0037 | 0.0369 |
|  | “Mainly private” - “Work only” | -2.83 | 0.0023 | 0.0023 | 0.0230 |
|  | “Mainly work” - “Work only” | -0.29 | 0.3866 | 0.3866 | 1.0000 |
|  | “Private only” - “Work only” | -2.82 | 0.0024 | 0.0024 | 0.0240 |
