# Supplementary material for: Smartphone Usage Patterns and Sleep Behavior in Demographic Groups: Retrospective Observational Study
Source: J Med Internet Res. 2025 Jul 3;27:e60423. doi: 10.2196/60423 (PMC12271961; doi:10.2196/60423)
Supplement: Multimedia Appendix 9 [file jmir_v27i1e60423_app9.docx]

Multimedia Appendix 9. Dunn's Test of Differences in Proportion of Days with Exceeding 6 Hours of Nocturnal Smartphone Inactivity

| Group Category | Comparison | Z Value | *P* Value Uncorrected | *P* Value Before | *P* Value Adjusted |
| --- | --- | --- | --- | --- | --- |
| **Gender** | |  |  |  |  |
|  | “Female” - “Male” | 4.36 | 0.0000 | 0.0000 | 0.0000 |
| **Age** | |  |  |  |  |
|  | “Less than 18 years” – “60 years or older” | -3.57 | 0.0002 | 0.0002 | 0.0011 |
|  | “Less than 18 years” - “18 years or older < 35 years” | -2.09 | 0.0182 | 0.0182 | 0.1094 |
|  | “60 years or older” - “18 years or older < 35 years” | 3.04 | 0.0012 | 0.0012 | 0.0071 |
|  | “Less than 18 years” - “35 years or older < 60 years” | -2.95 | 0.0016 | 0.0016 | 0.0096 |
|  | “60 years or older” - “35 years or older < 60 years” | 1.90 | 0.0285 | 0.0285 | 0.1712 |
|  | “18 years or older < 35 years” - “35 years or older < 60 years” | -3.90 | 0.0000 | 0.0000 | 0.0003 |
| **Highest degree** | |  |  |  |  |
|  | “Bachelor’s degree” - “Doctorate” | -1.12 | 0.1320 | 0.1320 | 1.0000 |
|  | “Bachelor’s degree” - “High school degree or equivalent” | -0.14 | 0.4426 | 0.4426 | 1.0000 |
|  | “Doctorate” - “High school degree or equivalent” | 1.10 | 0.1357 | 0.1357 | 1.0000 |
|  | “Bachelor’s degree” - “Master’s degree” | -2.83 | 0.0023 | 0.0023 | 0.0350 |
|  | “Doctorate” - “Master’s degree” | 0.04 | 0.4828 | 0.4828 | 1.0000 |
|  | “High school degree or equivalent” - “Master’s degree” | -3.19 | 0.0007 | 0.0007 | 0.0108 |
|  | “Bachelor’s degree” - “No formal qualification” | 1.92 | 0.0277 | 0.0277 | 0.4157 |
|  | “Doctorate” - “No formal qualification” | 2.21 | 0.0137 | 0.0137 | 0.2058 |
|  | “High school degree or equivalent” - “No formal qualification” | 1.99 | 0.0232 | 0.0232 | 0.3473 |
|  | “Master’s degree” - “No formal qualification” | 2.94 | 0.0017 | 0.0017 | 0.0248 |
|  | “Bachelor’s degree” - “Secondary education” | -2.02 | 0.0215 | 0.0215 | 0.3223 |
|  | “Doctorate” - “Secondary education” | 0.30 | 0.3819 | 0.3819 | 1.0000 |
|  | “High school degree or equivalent” - “Secondary education” | -2.21 | 0.0135 | 0.0135 | 0.2019 |
|  | “Master’s degree” - “Secondary education” | 0.60 | 0.2744 | 0.2744 | 1.0000 |
|  | “No formal qualification” - “Secondary education” | -2.66 | 0.0039 | 0.0039 | 0.0589 |
| **Employment status** | |  |  |  |  |
|  | “Full-time” - “Homemaker” | 0.16 | 0.4345 | 0.4345 | 1.0000 |
|  | “Full-time” - “In education” | 2.28 | 0.0113 | 0.0113 | 0.2363 |
|  | “Homemaker” - “In education” | 0.46 | 0.3226 | 0.3226 | 1.0000 |
|  | “Full-time” - “Part-time” | -0.80 | 0.2113 | 0.2113 | 1.0000 |
|  | “Homemaker” - “Part-time” | -0.45 | 0.3247 | 0.3247 | 1.0000 |
|  | “In education” - “Part-time” | -2.69 | 0.0035 | 0.0035 | 0.0742 |
|  | “Full-time” - “Retired” | -2.12 | 0.0169 | 0.0169 | 0.3552 |
|  | “Homemaker” - “Retired” | -1.59 | 0.0559 | 0.0559 | 1.0000 |
|  | “In education” - “Retired” | -2.85 | 0.0022 | 0.0022 | 0.0465 |
|  | “Part-time” - “Retired” | -1.75 | 0.0403 | 0.0403 | 0.8464 |
|  | “Full-time” - “Self-employed” | -0.31 | 0.3790 | 0.3790 | 1.0000 |
|  | “Homemaker” - “Self-employed” | -0.32 | 0.3759 | 0.3759 | 1.0000 |
|  | “In education” - “Self-employed” | -1.31 | 0.0950 | 0.0950 | 1.0000 |
|  | “Part-time” - “Self-employed” | 0.15 | 0.4402 | 0.4402 | 1.0000 |
|  | “Retired” - “Self-employed” | 1.62 | 0.0528 | 0.0528 | 1.0000 |
|  | “Full-time” - “Unemployed/job-seeking” | 2.28 | 0.0114 | 0.0114 | 0.2391 |
|  | “Homemaker” - “Unemployed/job-seeking” | 1.35 | 0.0881 | 0.0881 | 1.0000 |
|  | “In education” - “Unemployed/job-seeking” | 1.54 | 0.0612 | 0.0612 | 1.0000 |
|  | “Part-time” - “Unemployed/job-seeking” | 2.56 | 0.0052 | 0.0052 | 0.1089 |
|  | “Retired” - “Unemployed/job-seeking” | 3.21 | 0.0007 | 0.0007 | 0.0141 |
|  | “Self-employed” - “Unemployed/job-seeking” | 2.08 | 0.0190 | 0.0190 | 0.3981 |
