# Supplementary material for: Smartphone Usage Patterns and Sleep Behavior in Demographic Groups: Retrospective Observational Study
Source: J Med Internet Res. 2025 Jul 3;27:e60423. doi: 10.2196/60423 (PMC12271961; doi:10.2196/60423)
Supplement: Multimedia Appendix 10 [file jmir_v27i1e60423_app10.pdf]

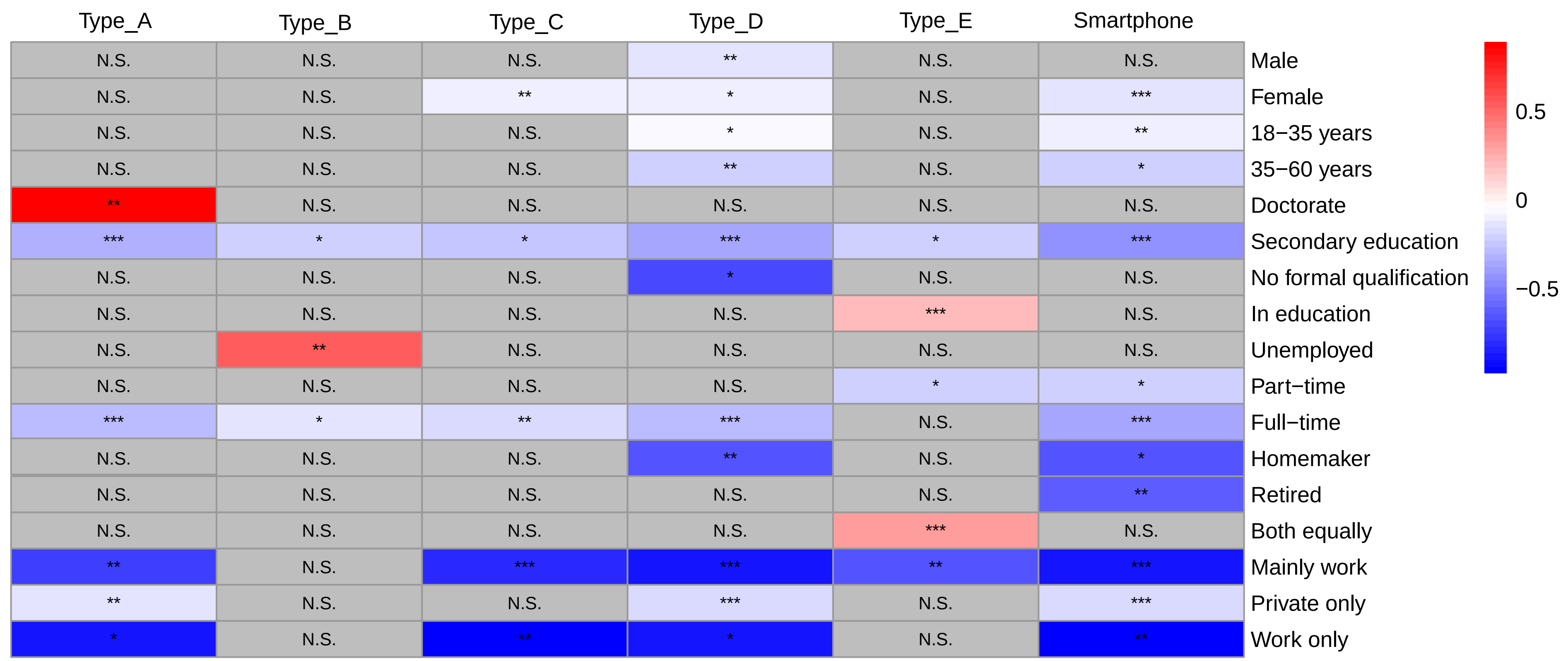

Multimedia Appendix 10. Correlation and Regression Heatmap: Linear Analysis of Daily Application Duration (Type A-E) and Daily Smartphone Usage in Relation to Nocturnal Inactivity Duration across Demographic Groups
