# Supplementary material for: Smartphone Usage Patterns and Sleep Behavior in Demographic Groups: Retrospective Observational Study
Source: J Med Internet Res. 2025 Jul 3;27:e60423. doi: 10.2196/60423 (PMC12271961; doi:10.2196/60423)
Supplement: Multimedia Appendix 11 [file jmir_v27i1e60423_app11.docx]

Multimedia Appendix 11. Correlation and Regression Analysis: Comparing Daily Duration between Smartphone Application Usage (Type A, B, C) and Nocturnal Smartphone Inactivity

| **Group Category** | | Type A | | | | | Type B | | | | | Type C | | | | |
| --- | --- | --- | --- | --- | --- | --- | --- | --- | --- | --- | --- | --- | --- | --- | --- | --- |
|  |  | Correlation Coefficient | Regression Coefficient | Regression Intercept | *p-*value | R-squared | Correlation Coefficient | Regression Coefficient | Regression Intercept | *p-*value | R-squared | Correlation Coefficient | Regression Coefficient | Regression Intercept | *p*-value | R-squared |
| **Gender (n=987)** | |  |  |  |  |  |  |  |  |  |  |  |  |  |  |  |
|  | Male(n=309) | -.07 | -.25 | 9.10 | .22 | .005 | .01 | .02 | 8.91 | .86 | .000 | .04 | .32 | 8.84 | .51 | .001 |
|  | Female(n=678) | -.07 | -.22 | 8.78 | .06 | .005 | -.07 | -.11 | 8.74 | .07 | .005 | -.10 | -.52 | 8.79 | .01 | .01 |
| **Age (n=987)** | |  |  |  |  |  |  |  |  |  |  |  |  |  |  |  |
|  | Less than 18 years (n=7) | .19 | 1.08 | 9.12 | .69 | .03 | -.35 | -.64 | 10.39 | .44 | .13 | -.03 | -.23 | 9.66 | .95 | .001 |
|  | 18 years or older < 35 years (n=820) | -.05 | -.18 | 8.80 | .13 | .003 | -.02 | -.03 | 8.71 | .58 | .000 | -.06 | -.44 | 8.80 | .08 | .004 |
|  | 35 years or older < 60 years (n=150) | -.13 | -.33 | 8.95 | .11 | .02 | -0.07 | -.09 | 8.79 | .42 | .004 | -.09 | -.27 | 8.84 | .28 | .008 |
|  | 60 years or older (n=10) | -.22 | -2.22 | 12.52 | .54 | .05 | -.44 | -1.58 | 12.33 | .20 | .20 | -.24 | -4.88 | 12.56 | .51 | .06 |
| **Highest degree (n=973)** | |  |  |  |  |  |  |  |  |  |  |  |  |  |  |  |
|  | Doctorate (n=10) | .89 | 2.98 | 7.14 | .002 | .74 | .20 | .21 | 8.80 | .59 | .04 | .45 | 7.99 | 7.48 | .19 | .20 |
|  | Master’s degree (n=106) | -.19 | -.68 | 9.55 | .06 | .03 | -.05 | -.13 | 9.08 | .63 | .002 | -.01 | -.08 | 9.02 | .91 | .000 |
|  | Bachelor’s degree (n=173) | -.03 | -.08 | 8.70 | .70 | .001 | -.06 | -.14 | 8.77 | .42 | .004 | -.09 | -.62 | 8.81 | .26 | .007 |
|  | Secondary education (n=90) | -.34 | -1.51 | 9.71 | < .001 | .11 | -.23 | -.35 | 9.30 | .03 | .05 | -.25 | -1.17 | 9.34 | .02 | .06 |
|  | High school degree or equivalent (n=584) | -.04 | -.13 | 8.73 | .36 | .001 | .007 | .01 | 8.63 | .87 | 4.4e-05 | -.04 | -.23 | 8.71 | .33 | .002 |
|  | No formal qualification (n=10) | -.40 | -3.43 | 10.53 | .26 | .16 | -.10 | -.22 | 9.56 | .78 | .01 | -.56 | -8.45 | 11.37 | .09 | .32 |
| **Employment status (n=960)** | |  |  |  |  |  |  |  |  |  |  |  |  |  |  |  |
|  | In education (n=485) | .03 | .11 | 8.58 | .48 | .001 | -.03 | -.05 | 8.72 | .51 | .001 | .003 | .02 | 8.65 | .95 | 8.8e-06 |
|  | Unemployed job-seeking (n=20) | -.13 | -.56 | 10.39 | .60 | .02 | .55 | .61 | 8.67 | .01 | .30 | -.05 | -.25 | 10.09 | .84 | .002 |
|  | Part-time (n=136) | -.10 | -.31 | 8.99 | .23 | .01 | -.12 | -.35 | 9.02 | .16 | .02 | -.06 | -.38 | 8.88 | .49 | .04 |
|  | Full-time (n=256) | -.27 | -.69 | 8.96 | < .001 | .07 | -.15 | -.20 | 8.68 | .02 | .02 | -.17 | -.68 | 8.72 | .006 | .03 |
|  | Self-employed (n=34) | .27 | 1.19 | 8.57 | .12 | .07 | -.07 | -.23 | 9.40 | .69 | .005 | -.03 | -.17 | 9.35 | .88 | .001 |
|  | Homemaker (n=13) | -.30 | -2.52 | 10.83 | .33 | .09 | -.33 | -.50 | 10.04 | .27 | .11 | -.52 | -7.05 | 11.23 | .07 | .27 |
|  | Retired (n=16) | -.23 | -2.48 | 11.97 | .38 | .05 | -.45 | -3.75 | 11.94 | .08 | .21 | -.43 | -6.89 | 12.46 | .10 | .18 |
| **Smartphone use type (n=987)** | |  |  |  |  |  |  |  |  |  |  |  |  |  |  |  |
|  | Both equally (n=124) | .05 | .21 | 8.74 | .57 | .003 | .06 | .11 | 8.77 | .51 | .004 | -.02 | -.14 | 8.94 | .81 | .001 |
|  | Mainly private (n=364) | .03 | .08 | 8.57 | .60 | .001 | .04 | .07 | 8.56 | .42 | .002 | -.05 | -.31 | 8.73 | .33 | .003 |
|  | Mainly work (n=13) | -.74 | -10.09 | 16.15 | .004 | .55 | -.55 | -18.47 | 14.60 | .05 | .30 | -.83 | -18.54 | 16.11 | < .001 | .69 |
|  | Private only (n=481) | -.13 | -.38 | 8.82 | .003 | .02 | -.06 | -.09 | 8.67 | .16 | .002 | -.02 | -.11 | 8.61 | .63 | .001 |
|  | Work only (n=5) | -.88 | -3.06 | 14.49 | .05 | .78 | -.88 | -16.04 | 15.28 | .05 | .77 | -.98 | -37.37 | 16.32 | .005 | .95 |

Note: In this table, the correlation coefficient measures the strength and direction of the relationship between two variables. The regression coefficient indicates the magnitude of the impact of one variable on another, and the regression intercept represents the expected mean value of the dependent variable when all independent variables are zero. The *p*-value assesses the probability of observing the results assuming the null hypothesis is true, while the R-squared value indicates the proportion of variance in the dependent variable that can be explained by the independent variable.
